# Harnessing Novel Diversity From Landraces to Improve an Elite Barley Variety

**DOI:** 10.3389/fpls.2019.00434

**Published:** 2019-04-11

**Authors:** Arantxa Monteagudo, Ana M. Casas, Carlos P. Cantalapiedra, Bruno Contreras-Moreira, María Pilar Gracia, Ernesto Igartua

**Affiliations:** ^1^Aula Dei Experimental Station (EEAD-CSIC), Zaragoza, Spain; ^2^Fundación ARAID, Zaragoza, Spain

**Keywords:** barley, landrace, QTL, adaptation, 50k

## Abstract

The Spanish Barley Core Collection (SBCC) is a source of genetic variability of potential interest for breeding, particularly for adaptation to Mediterranean environments. Two backcross populations (BC_2_F_5_) were developed using the elite cultivar Cierzo as the recurrent parent. The donor parents, namely SBCC042 and SBCC073, were selected from the SBCC lines due to their outstanding yield in drought environments. Flowering time, yield and drought-related traits were evaluated in two field trials in Zaragoza (Spain) during the 2014–15 and 2015–16 seasons and validated in the 2017–18 season. Two hundred sixty-four lines of each population were genotyped with the Barley Illumina iSelect 50k SNP chip. Genetic maps for each population were generated. The map for SBCC042 × Cierzo contains 12,893 SNPs distributed in 9 linkage groups. The map for SBCC073 × Cierzo includes 12,026 SNPs in 7 linkage groups. Both populations shared two QTL hotspots. There are QTLs for flowering time, thousand-kernel weight (TKW), and hectoliter weight on a segment of 23 Mb at ~515 Mb on chromosome 1H, which encompasses the *HvFT3* gene. In both populations, flowering was accelerated by the landrace allele, which also increased the TKW. In the same region, better soil coverage was contributed by SBCC042 but coincident with a lower hectoliter weight. The second large hotspot was on chromosome 6H and contained QTLs with wide intervals for grain yield, plant height and TKW. Landrace alleles contributed to increased plant height and TKW and reduced grain yield. Only SBCC042 contributed favorable alleles for “green area,” with three significant QTLs that increased ground coverage after winter, which might be exploited as an adaptive trait of this landrace. Some genes of interest found in or very close to the peaks of the QTLs are highlighted. Strategies to deploy the QTLs found for breeding and pre-breeding are proposed.

## Introduction

Increasing crop yields is the main breeding target for cereals. This goal will become increasingly challenging in areas where the occurrence of limiting factors is expected to rise due to climate change, such as Mediterranean Europe. Due to their adaptability to a wide range of conditions, barley landraces are recognized as an important genetic resource with which to search for tolerance to biotic and abiotic stresses (Dawson et al., 2015). However, landrace potential has not been fully realized in modern breeding (Fischbeck, 2003; Langridge and Waugh, 2019). This fact was confirmed for wheat in a thorough study of a worldwide landrace collection with high throughput genotyping platforms (Winfield et al., 2018), revealing a substantial amount of novel genetic diversity in the landraces, which is either not captured in current breeding programs or lost due to previous selection pressures. The high diversity found in barley genetic resources predicts a similar situation for this crop (for instance, IBSC, 2012; Russell et al., 2016), or even a more diverse one, as the barley genome has been enriched by protracted gene flow between the cultivated and wild species for thousands of years (Poets et al., 2015).

Barley landraces are valuable resources for breeding in the Mediterranean region (Ceccarelli et al., 1998; Comadran et al., 2009), given their long history of selection under stressful conditions. For instance, landraces out yielded modern varieties in different studies carried out in Syria (Ceccarelli, 1996) and in Spain (Yahiaoui et al., 2014) when grown in harsh to moderate stress conditions. The current study focuses on the Spanish Barley Core Collection (SBCC), which is a powerful tool with which to study and apply the adaptive potential of Spanish landraces to Mediterranean conditions (Igartua et al., 1998). The SBCC accessions contain unique alleles compared to the barley genotypes used in mainstream barley breeding in Europe, particularly in the six-rowed barley pool (Yahiaoui et al., 2008). The accessions also carry adaptations to biotic stresses (Silvar et al., 2010) and to environmental conditions that may be useful in a climate change scenario (Casas et al., 2011). On the negative side, SBCC landraces tend to be tall plants, with late flowering and a risk of lodging (Yahiaoui et al., 2014). In this last study, the landrace-derived lines SBCC073 and SBCC042 were among the top 5% highest-yielding lines in field trials with average yields below 3 t ha^−1^.

In the past, one of the reasons for the limited use of landraces to introduce new genetic variation into breeding programs was linkage drag. Currently, the advent of new platforms of molecular markers provides a solution to overcome this problem (Muñoz-Amatriaín et al., 2014; Russell et al., 2016; Milner et al., 2019), and the use of these platforms is becoming routine in crop breeding programs (Trevaskis, 2018). The new 50k Illumina Infinium iSelect SNP genotyping array (Bayer et al., 2017) will facilitate precise access to the genomic diversity of the landraces and its efficient use in breeding programs.

The most important trait in agriculture is yield, but it is a complex breeding trait due to its low heritability, pleiotropic effects, and susceptibility to genotype-by-environment (G × E) interaction. A large G × E component has hampered breeding progress in the Mediterranean region in the past (Muñoz et al., 1998), and this situation is expected to only intensify in the near future, given the predictions of climate models (Trnka et al., 2011). The strategy for improving crop yield requires selection of its best genetic component, through the contribution of well-known individual or combined alleles. Some of the combinations that lead to large yield improvements in crops are associated with plant height and flowering time (Cockram et al., 2007; Nadolska-Orczyk et al., 2017). Short cereals exhibited improved grain production and a lower risk of lodging. An optimized flowering time allows plants to benefit from rainfall at early stages and better grain–filling conditions. These features are usually fine-tuned for optimum performance of elite germplasm in each region. A sensible strategy for plant breeding would be to introgress good adaptive features from landraces into elite germplasm developed locally. Judicious selection of parents could lead to candidate cultivars in a rapid manner.

One possible advantage of landraces over modern cultivars in Mediterranean environments is their enhanced early growth vigor. This trait was found in Mediterranean landraces from areas with mild winters (Van Oosterom and Acevedo, 1992) and was identified as one of the factors associated with increased yield under drought (Turner and Nicholas, 1998). A survey of barley varieties obtained over a 100 years of breeding in Nordic countries revealed an overall decrease in early vigor (root and shoot). This decrease was explained by the introduction of semidwarf genes, which increased the harvest index and reduced lodging, and adaptation to agriculture with high fertilizer application (Bertholdsson and Kolodinska-Brantestam, 2009). Early vigor is positively correlated with grain yield and drought tolerance in cereals (Ludwig and Asseng, 2010; Pang et al., 2014). Earlier studies (Van Oosterom and Acevedo, 1992) found that high early vigor was related to good yield in Mediterranean environments, but only in landraces from areas with mild winters, whereas the opposite was true for landraces from areas with colder winters. A delicate balance between water availability, early development and cold tolerance must be achieved to optimize grain yield.

The objectives of this study are: i) to find QTLs for agronomic traits in elite-by-landrace crosses; ii) to evaluate the feasibility of improving an elite cultivar with introgressions from two local landraces, that have shown high performance under low productivity conditions; and iii) to assess the usefulness of RGB imaging during early growth, and its relation to grain yield. The positive alleles contributed by landraces could be directly used in breeding to improve elite cultivars. Positive alleles contributed by the elite cultivar will indicate genomic regions of landraces that could be targeted by pre-breeding programs to improve key landrace features.

## Materials and Methods

### Plant Materials

Two barley BC_2_F_5_ populations were developed from crosses between Cierzo and two Spanish landrace-derived inbred lines from the SBCC (Igartua et al., 1998) (((landrace × Cierzo) × Cierzo) × Cierzo). The recurrent parent, Cierzo is an elite six-rowed barley cultivar derived from the cross Orria × Plaisant and selected in Spain. The parent is high-yielding, with an intermediate growth habit and good malting quality, although it is relatively less productive in arid zones.^1^ The donor parents were SBCC042 and SBCC073, both of which are six-rowed and high-yielding in low-productivity trials and have an intermediate growth habit (Yahiaoui et al., 2014). After two backcrosses, 270 BC_2_F_2_ lines of each population were selfed for three generations, up to BC_2_F_5_. After a few losses, we derived 264 BC_2_F_5_ lines of each advanced cross. The trial was sown following a type II augmented design (Lin and Poushinsky, 1985). The lines were tested in two field trials carried out in the province of Zaragoza in northwestern Spain (41°51′ N, 0°39′ W) in the 2014–2015 and 2015–2016 seasons. The trials were sown in autumn (November 10th, 2014, and November 12th, 2015). The cultivars Cierzo and Orria were used as main checks, with a replicate in each incomplete block of 12 plots, for a total of 28 replicates per check and population. The secondary checks were the donor parent and Plaisant cultivar, randomly repeated in 8 blocks. The plots consisted of four rows that were 3.0 m long × 0.8 m wide. Climatic data were provided by the Spanish Meteorology State Agency (AEMET) and were gathered from a station in the same location as the trials (Zuera) (Figure S1).

A sample of 96 lines of the population SBCC073 × Cierzo was trialed again in the field in the 2017–2018 season at the same location, using the same plot size, two replicates, and a randomized complete block design. These lines were selected for homogeneous flowering dates; therefore, the earliest and latest lines were culled. The aboveground biomass of plants in two contiguous rows (25 cm per row, 50 cm in total, 0.10 m^2^) of a representative zone of the plot was hand-harvested at ground level, and used to estimate yield components and the harvest index. This trial was used to validate the QTLs found in the previous seasons.

### Phenotyping

Plots were scored for grain yield (GY), plant height (PH), flowering date (FD), thousand- kernel weight (TKW), hectoliter weight (HW), crop cover as green area (GA) or greener area (GGA) and SPAD (chlorophyll content measured with soil plant analysis development) (Table S1). The plots were combine-harvested, and grain yield was converted to kg ha^−1^, taking into account the harvested area per plot. Plant height was measured in cm from the soil to the base of the spike at maturity in one representative plant per plot. Flowering time was recorded as the number of days from January 1st until the date when 50% of the stems of each plot displayed 2-cm protruding awns (stage 49, Zadoks scale, Zadoks et al., 1974). Hectoliter weight (kg hl^−1^) was estimated with a grain analyzer model GAC- II (Dickey-John, USA) by measuring the weight of a constant volume. Thousand-kernel weight (g) was calculated from the weight of a 1000-grain sample. Green and greener areas were measured with zenithal pictures of each plot and analyzed using Breedpix software (Casadesús and Villegas, 2014). These indexes are estimates of the ground cover at the end of the vegetative period and of the early vigor of the lines, according to crop development on the dates when the photos were taken (March 13th, 2015 and February 17th, 2016). A single digital picture was taken with a Nikon Coolpix B700 camera held 145–150 cm above the ground and in front of the sun to avoid shading. Each picture contained the four rows of a single plot, focusing on the center of the plot. The zoom was set to an 8 mm focal length with a semiautomatic aperture, prioritizing the shutter speed, which was adjusted to 1/125 s. These parameters help avoid problems caused by wind and hand movements. SPAD color measurements were taken during May 10–11th, 2016. Ten measurements per plot were taken from the flag leaves of 10 randomly chosen plants (2–3 per row) with a SPAD chlorophyll meter (SPAD-502, Minolta, Japan).

Soil variation was observed in the two dimensions of the trial. Therefore, to minimize error due to autocorrelation among adjacent plots, raw data were spatially corrected in both directions using a moving average correction approach in R (R Core Team, 2014) with the mvngGrAd R package (Technow, 2011), as in Nice et al. (2017). This procedure performs a correction similar to the augmented design, optimizing the grid size and shape used for adjustment in two dimensions and searching the moving average grid by minimizing the variance in the primary and secondary checks. To validate the procedure, two calculations were performed: (1) the correlation between the 2 years and (2) Pearson's coefficient of variation (CV) between the testers for each year. If the correlation between the adjusted values was greater than the correlation between the observed values and the adjusted values' CV was lower than the observed values' CV, we considered the data to be well-adjusted.

Principal component analyses (PCAs) were performed with the function PCA in the R package FactoMineR 1.41 (Lê et al., 2008). Correlation analyses were performed using the R package corrplot 0.84 (Wei and Simko, 2017).

### Genotypic Analysis and Map Construction

Genomic DNA was obtained from one leaf per genotype of 10-days-old plants using a NucleoSpin® Plant II kit (Macherey-Nagel, Germany). DNA concentration was quantified using a Nanodrop 2000 (Thermo Scientific, USA).

A total of twelve 48-well plates of the Barley 50k iSelect SNP Array (Bayer et al., 2017) were processed by the CEGEN service, *Centro Nacional de Investigaciones Oncológicas* (CNIO, Spain). This chip scores 44,040 SNPs. The parents, an artificial F_1_ (DNA mixture of the two parents), and the cultivar Morex were included in each plate as controls. SNP alleles were called using GenomeStudio Genotyping Module v2.0.2 (Illumina, USA). Calling was manually curated as recommended by Bayer et al. (2017). At any single marker, the average theoretical homozygosity should be 98.44%. Therefore, true heterozygotes should appear at a frequency of 1.56%. Given the type of population, the expected segregation was 7:1 (7 Cierzo alleles per allele from SBCC042 or SBCC073). Markers with too many missing data (call frequency < 0.7) and excess of heterozygotes (Het_Excess_Freq > −0.6) were filtered out, as were monomorphic markers. The resulting data were loaded into Flapjack (Milne et al., 2010) for visual inspection of graphical genotypes. Segregating SNPs were grouped into linkage groups with a LOD score of 8 with JoinMap 4.0 (Van Ooijen, 2006). Cosegregating SNPs were excluded to increase computing efficiency. The maximum likelihood algorithm was used to estimate the best order of markers within each linkage group. The final map order was computed in the R/qtl package (Arends et al., 2010). The distance between the markers was calculated based on Kosambi's mapping function using the Viterbi's algorithm with the function *quickest* in ASMAP (Taylor and Butler, 2016). The resulting maps were matched to the position in the reference genome (Mascher et al., 2017) using *compareOrder*, aiming to maintain high LOD scores while keeping the map length to a minimum. Segregation distortion was calculated with the *geno.table* function (R/qtl). Then, cosegregating SNPs were included again in the dataset.

### QTL Analysis

QTL analyses were performed in GenStat 18 (Payne et al., 2011) using the single-trait linkage analysis procedure (single or multiple environments, as appropriate). Genetic predictors were estimated every 2 cM. In the first step, simple interval mapping (SIM) was run. Then, detected QTL were used as cofactors to carry out the second step, running a composite interval mapping (CIM). Following CIM, rounds were run until a stable solution was found. The Li and Ji (2005) method was used to estimate the significance threshold of –log_10_P to declare putative QTLs with an overall significance level of 0.05. The minimum distance between cofactors was established as 15 cM, and the minimum distance between QTLs was set to 10 cM. In the last step, the final QTL model was built. Confidence intervals (95% Bayes credible intervals) for the QTLs were calculated in R/qtl using function *Bayesint*. Genomic regions surrounding the QTL were explored, taking into account the genes between the markers cosegregating at each peak, according to positions reported by BARLEYMAP (Cantalapiedra et al., 2015); then, all genes within the confidence intervals were retrieved. Possible interactions between QTLs were examined with pairwise analyses of variance in the marker peaks for each trait. Venn diagrams were computed and plotted with R, using the package “VennDiagram” (Chen and Boutros, 2011). MapChart (Voorrips, 2002) was used to represent genetic maps and QTL positions. Physical maps combining information obtained for both populations were constructed with the R package “ggplot2” (Wickham, 2016).

## Results

Spatial corrections were based on finding the optimum surrounding grid per trait, as in Nice et al. (2017), which resulted in 4 plots on each side in the horizontal direction and 2 plots per side in the vertical direction. Each plot was adjusted according to a grid of 24 neighboring plots (8 in the plot's row and 5 and 3 in the row above and below) and a minimum of 9 plots at the corners of the experiment.

**Figure 1:**
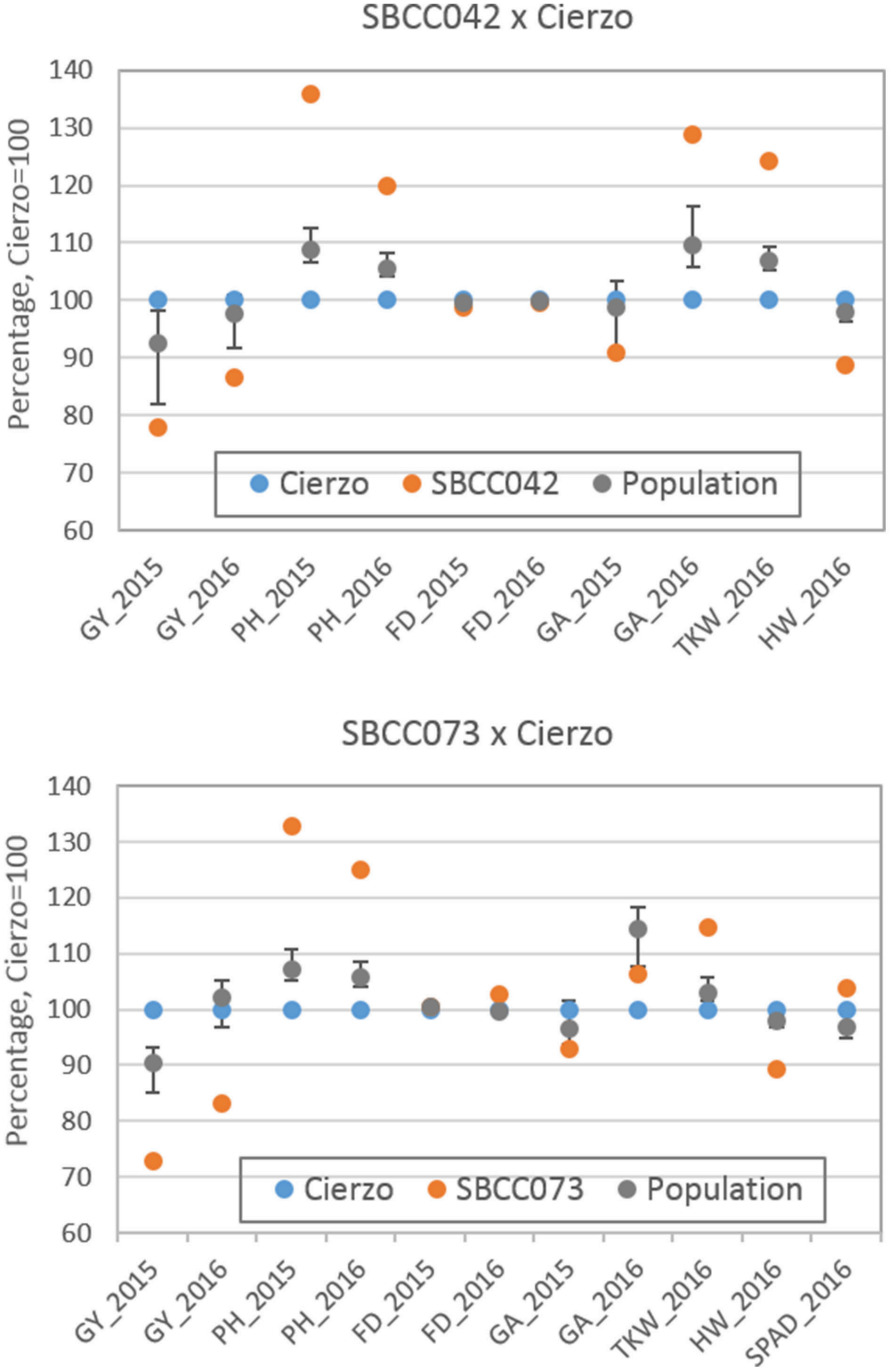
Schematic representation of trait averages for the two BC_2_F_5_ barley populations, compared to those of their parents, showing the least significant differences (*P* = 0.05) of the population average against each parent. The elite parent Cierzo was given a value of 100 for each trait, and other averages and LSDs were calculated relative to Cierzo.

The three seasons were different regarding yield levels. The first season, 2015, was a rather typical year, with terminal drought and yields of ~3 t ha^−1^. In the second season, however, the yields ranged between 5 and 6 t ha^−1^ for most accessions, remarkably high production for the test site. The validation season was similar to 2015–16, although with lower overall yields, despite abundant precipitation in spring (Figure S1). As expected, the populations' averages were, in general, intermediate between the parents, but much closer to the elite parent, as it contributed 87.5% of the genome of each population (Tables 1, 2). On average, the landrace parents yielded 20% less than Cierzo, the elite parent, whereas the populations yielded only 4% less. There were remarkable disparities among years for these differences. In the less productive year, 2015, the yield of both landrace parents and the population averages were more distant from the yield of the elite parent than in 2016 (Figure 1). There was a large difference between Cierzo and the landrace parents in PH (15–22 cm), TKW (1.5–9 g) and HW (7–8 kg hl^−1^). For these traits, the population means fell between the parental values but closer to that of the elite parent. All parents exhibited similar flowering dates, with a maximum of 3 days between extremes in any year. For SBCC042 × Cierzo, the flowering dates for the parents and the population mean were very similar. For SBCC073 × Cierzo, the landrace parent flowered slightly later than Cierzo. The population mean was closer to the value of the landrace parent and even outside the interparental range in 2016. Regarding ground cover during vegetative growth, the parents exhibited a contrasting pattern among years. Both landraces displayed values lower than that of Cierzo in 2015 and higher than that in Cierzo in 2016 (although at an earlier stage), with all but one population at intermediate positions. For SBCC042 × Cierzo, the population means were always within the interparental range. In contrast, in SBCC073 × Cierzo, some traits exhibited population means outside the interparental range. This discrepancy cannot be explained by a purely additive model. There must have been some epistatic genes whose effects were distorted in the crossing. Such a pattern occurred for the SPAD score and the GA in 2016. In these cases, the population mean was significantly different from the values of both parents. Interestingly, the average GY of the population in 2016 was also higher than both parents, although not significantly. The population means were based on measurements from 200 plots, while the elite parent mean was the average from 28 plots and the landrace parent mean was from 8 plots per year. Therefore, the estimates of these values were quite robust.

Cierzo exhibited agronomic advantages in terms of PH, HW and TKW. We expected the landraces, and their populations to perform better than the elite parent in the drier, less productive year, but we found the opposite pattern. We must highlight that Cierzo, a successful cultivar in recent years, was bred in Spain, and the location used for the current experiment was among those used to select this cultivar. Therefore, we tested some of the best possible landraces against one of the best modern cultivars for the region. The landraces and the elite line presented remarkable differences in GY, PH, TKW and HW. Cierzo out yielded the landraces in both years, having a greater HW and a shorter stature (Tables 1, 2). The landraces, however, presented a much higher TKW than the elite parent (Tables 1, 2). Differences between genotypes were significant for GY, PH, and FD in both populations, as shown by the ANOVA results (Table S2), which were calculated with the residuals provided by the replicated checks. Both populations showed normal distributions and transgressive segregation for most traits, with some exceptions, such as HW, whose distribution was nearly bimodal (Figures S2, S3). Among the ground cover traits, namely, GA and GGA, both parents showed similar covertures in March 2015, and SBCC042 showed more coverture than Cierzo in February 2016 (Figure S3a; Table 1). Few differences were found between SBCC073 and Cierzo in both traits and in SPAD measurements (Figure S3b; Table 2).

**Table 1:** Trait averages for the SBCC042 × Cierzo population, parents, and checks.

**Traits**	**Population**			**Cierzo**			**SBCC042**			**Orria**			**Plaisant**		
	**Year**	**Mean**	**SD**	**Mean^#^**	**SD**		**Mean^#^**	**SD**		**Mean^#^**	**SD**		**Mean^#^**	**SD**	
Grain yield (t ha^−1^)	2015	3.45	1.07	3.71	0.89	a	2.89	0.89	b	3.22	0.92	b	1.36	0.54	c
	2016	5.79	0.93	5.92	0.94	a	5.12	0.82	b	5.28	1.11	b	4.04	0.86	c
Plant height (cm)	2015	67.1	7.54	61.77	6.90	c	83.9	3.45	a	55.5	5.36	d	77.4	4.66	b
	2016	79.8	5.91	75.51	5.35	c	90.5	3.82	a	72.2	5.51	d	83.9	5.52	b
Flowering date (Julian days)	2015	106.9	2.61	107.3	1.24	a	105.8	1.61	b	106.2	2.00	b	108.5	0.85	a
	2016	112.4	2.47	112.5	1.37	b	112.0	0.82	b	113.1	1.85	b	114.8	1.31	a
GA March	2015	0.57	0.15	0.58	0.13	a	0.52	0.15	ab	0.47	0.12	b	0.56	0.10	ab
GGA March	2015	0.43	0.15	0.43	0.13	a	0.39	0.15	ab	0.34	0.12	b	0.40	0.10	ab
TKW (g)	2016	40.1	3.17	37.5	1.37	b	46.6	2.65	a	37.1	2.55	b	37.3	1.60	b
HW (kg hl^−1^)	2016	67.2	2.91	68.7	1.78	a	60.9	1.64	c	65.6	1.51	b	69.5	1.43	a
GA Feb	2016	0.34	0.07	0.31	0.07	ab	0.40	0.04	c	0.24	0.05	b	0.23	0.02	a
GGA Feb	2016	0.24	0.06	0.21	0.06	ab	0.30	0.04	b	0.15	0.04	b	0.15	0.02	a
GA ratio	2016	0.63	0.30	0.54	0.18	b	1.05	0.35	a	0.51	0.28	b	0.36	0.17	b

# *The difference between means followed by the same letter is not significantly larger than the LSD (P < 0.05)*.

*GA, green area; GGA, greener area; TKW, thousand-kernel weight; HW, hectoliter weight*.

**Table 2:** Traits averages for the SBCC073 × Cierzo population, parents and checks.

**Traits**	**Population**			**Cierzo**			**SBCC073**			**Orria**			**Plaisant**		
	**Year**	**Mean**	**SD**	**Mean^#^**	**SD**		**Mean^#^**	**SD**		**Mean^#^**	**SD**		**Mean^#^**	**SD**	
Grain yield (t ha^−1^)	2015	4.24	0.65	4.70	0.59	a	3.42	0.51	c	3.98	0.64	b	1.61	0.56	d
	2016	5.99	0.92	5.88	0.74	a	4.89	0.49	b	5.04	0.63	b	3.60	0.88	c
Plant height (cm)	2015	67.6	7.28	62.9	5.49	c	83.6	3.98	a	59.4	6.01	d	71.9	4.78	b
	2016	80.9	6.54	76.4	4.96	c	95.4	7.55	a	70.8	4.76	d	81.5	3.94	b
Flowering date (Julian days)	2015	107.9	2.25	107.6	1.12	a	108.1	0.92	a	108.0	1.79	a	108.4	0.77	a
	2016	110.4	1.94	110.92	1.00	c	113.8	1.96	a	110.5	1.71	c	112.2	1.12	b
GA March	2015	0.77	0.11	0.80	0.09	a	0.74	0.09	ab	0.65	0.10	c	0.66	0.07	bc
GGA March	2015	0.67	0.13	0.70	0.11	a	0.64	0.09	a	0.54	0.10	b	0.53	0.08	b
TKW (g)	2016	39.6	3.06	38.4	1.99	b	44.1	1.81	a	35.3	2.32	c	35.4	1.61	c
HW (kg hl^−1^)	2016	67.3	2.37	68.8	1.13	a	61.5	2.75	c	64.7	2.10	b	68.3	0.91	a
SPAD	2016	38.3	3.41	39.6	2.91	a	41.1	3.03	a	36.4	2.95	b	41.8	2.44	a
GA Feb	2016	0.33	0.07	0.29	0.05	a	0.31	0.04	a	0.24	0.04	b	0.21	0.03	c
GGA Feb	2016	0.23	0.06	0.20	0.04	a	0.22	0.04	a	0.15	0.04	b	0.13	0.03	b
GA ratio	2016	0.65	0.33	0.52	0.16	b	1.16	0.48	a	0.49	0.20	b	0.36	0.13	b

# *The difference between means followed by the same letter is not significantly larger than the LSD (P < 0.05)*.

*GA, green area; GGA, greener area; TKW, thousand-kernel weight; HW, hectoliter weight*.

### Correlations Between Traits

Negative correlations were observed between GY and FD; in other words, later flowering was associated with decreased yield (Figures S4–S8). Water stress during grain filling is a common occurrence in Mediterranean climates; therefore, this correlation was not unexpected. We observed positive correlations between GY and PH in most cases (SBCC073 × Cierzo in 2015 was the exception). Overall, these two traits showed moderate and positive correlations with ground cover in both populations and years, particularly in SBCC042 × Cierzo. The positive correlation between PH and GY is problematic from a plant breeding point of view. Spanish barley landraces are usually taller than modern cultivars, and one of the reasons for the replacement of the former was their susceptibility to lodging in current high-input agriculture (Yahiaoui et al., 2014). The most straightforward way to convert the landraces into modern feed cultivars would be to reduce their height without incurring a yield penalty. This goal is probably unachievable with these biparental crosses. Height reduction through marker-assisted selection, and possibly even through genomic selection, would not be enough to achieve the same height as that of the elite parent, given the antagonistic correlation between yield and height, which is at least partially caused by the QTL in 6H shared by the two populations.

Both populations demonstrated a significant contribution of GY to the first dimension of the biplots based on correlations (Figure S9), which was almost orthogonal with PH in 2015. In contrast, both parameters were strongly correlated in 2016, possibly because of the good conditions experienced in that year. Flowering contributed substantially to the second dimension in 2015 and was responsible of the major differences in dimension 1 in 2016, but with a negative sign. This result can also be explained by the different climatic conditions experienced in both years. While 2015 was a normal-to-low year in terms of spring rainfalls and temperatures, 2016 was an excellent year with a very humid and mild spring. In all cases, coverture at early stages significantly contributed to the first dimension. GA at early stages (2016) or advanced vegetative growth (2015) was related to early flowering and taller plants. HW and SPAD (only recorded in 2016) were highly correlated with late flowering that year, and a higher TKW was obtained in lines with more coverture at early stages in SBCC073 × Cierzo, with early flowering in both cases.

### Genetic Map

Among the 44,040 markers, 12,893 and 12,026 SNPs were polymorphic and of high quality in SBCC042 × Cierzo and SBCC073 × Cierzo, covering total distances of 1,080.6 and 1,115.8 cM, respectively (Table S3 and Supplementary Data 1). Landrace alleles provided good coverage of the whole genome (graphical genotypes, Figure S10). After quality filtering, 206 and 241 lines were kept for the QTL analysis, for SBCC042 × Cierzo and SBCC073 × Cierzo, respectively. In all instances in which we refer to the two populations, SBCC042 × Cierzo will be reported first and SBCC073 × Cierzo last. In these populations, 987 and 875 markers corresponded to unique genetic positions (one marker in one position among all the cosegregating markers) in the two maps, respectively. Nine and seven linkage groups were identified in the populations. In SBCC042 × Cierzo, the 9 linkage groups represented 5 complete chromosomes (1H, 3H, 4H, 5H, and 7H) and 2 fragmented ones (2 groups each for 2H and 6H). One linkage group per chromosome was found in SBCC073 × Cierzo. The number of markers per chromosome ranged between 1,290 (1H) and 2,275 (5H) and between 1,273 (1H) and 2,087 (5H) in each population (Table S3). The average spacing between markers for each map was 1.0 and 1.3 cM, with maximum spacings of 18.2 and 15.1 cM (Table S3). In these BC_2_F_5_ populations, the expected percentages of allelic frequencies of elite and landrace parents were ~87.5:12.5. The actual frequencies were 83.7 AA: 2.1 AB: 14.2 BB and 86.2 AA: 1.7 AB: 12.1 BB (Figure S11). The populations shared 8,036 polymorphic markers (Figure S12a), which were mainly distributed in the distal and interstitial regions of the chromosomes (Figure S12b), except on chromosome 7H, where they also exhibited proximal and centromeric positions. Among the rest of the markers (4,857 in SBCC042 × Cierzo and 3,990 in SBCC073 × Cierzo), chromosome 2H showed specific markers for the SBCC073 × Cierzo population and distal part of the short arm in 5H showed a concentration of markers for SBCC042 × Cierzo. This last region showed some segregation distortion, with overrepresentation of Cierzo alleles in SBCC042 × Cierzo (Figure S11).

### QTL Analysis

**Figure 2:**
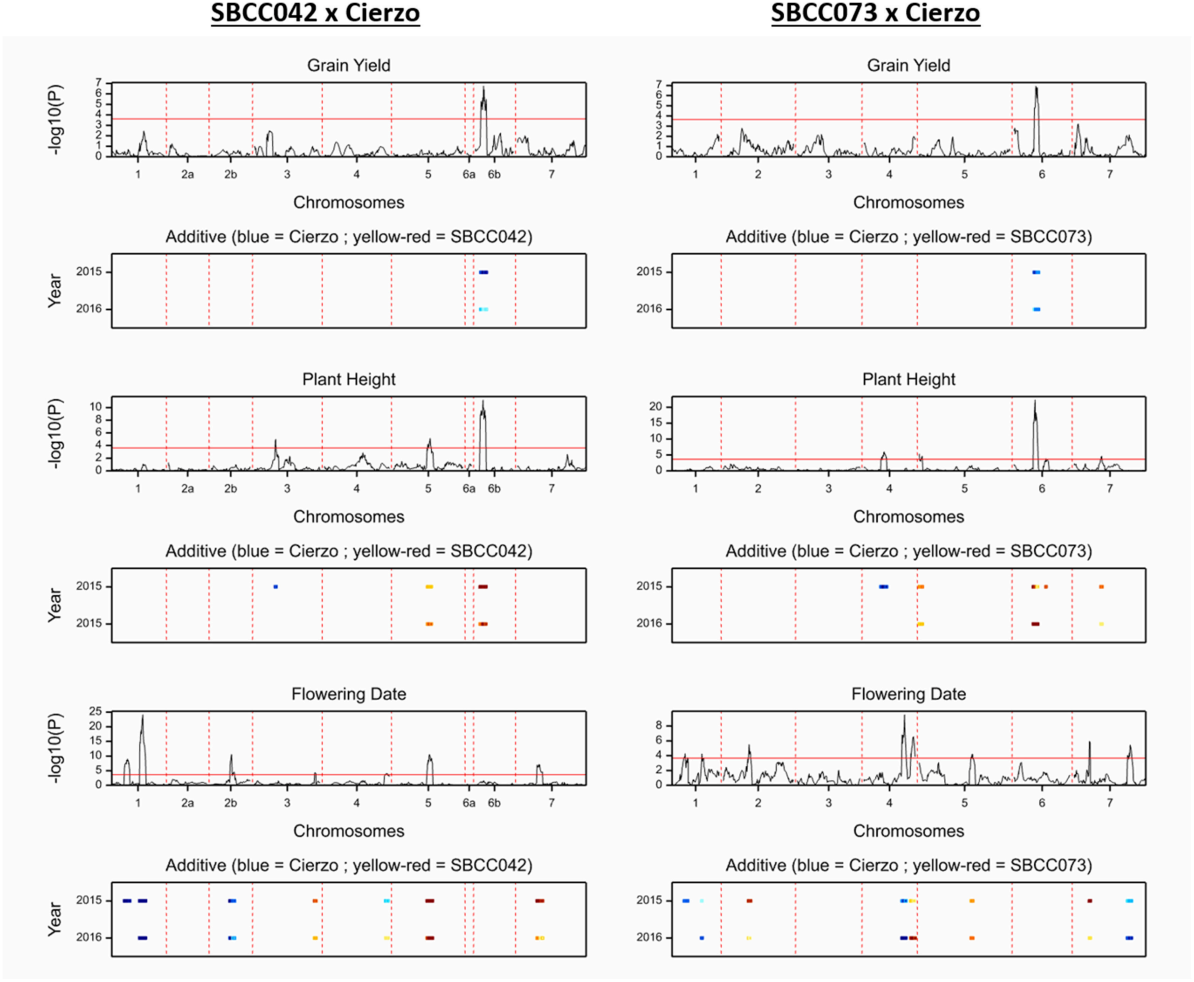
QTL scans of the whole genome for the traits measured in two seasons: grain yield, plant height and flowering date, from top to bottom; SBCC042 × Cierzo on the left, and SBCC073 × Cierzo is on the right. For each trait, the top graph represents the test statistic (–log10 of the *P*-value) against genetic distance (cM) for the seven chromosomes. The bottom graph indicates the width of the QTL peak in each season, and the color indicates the strength and direction of the effect: blue shades indicate that Cierzo is the trait increasing allele, and yellow to red shades indicate that the landrace allele increases the trait.

**Figure 3:**
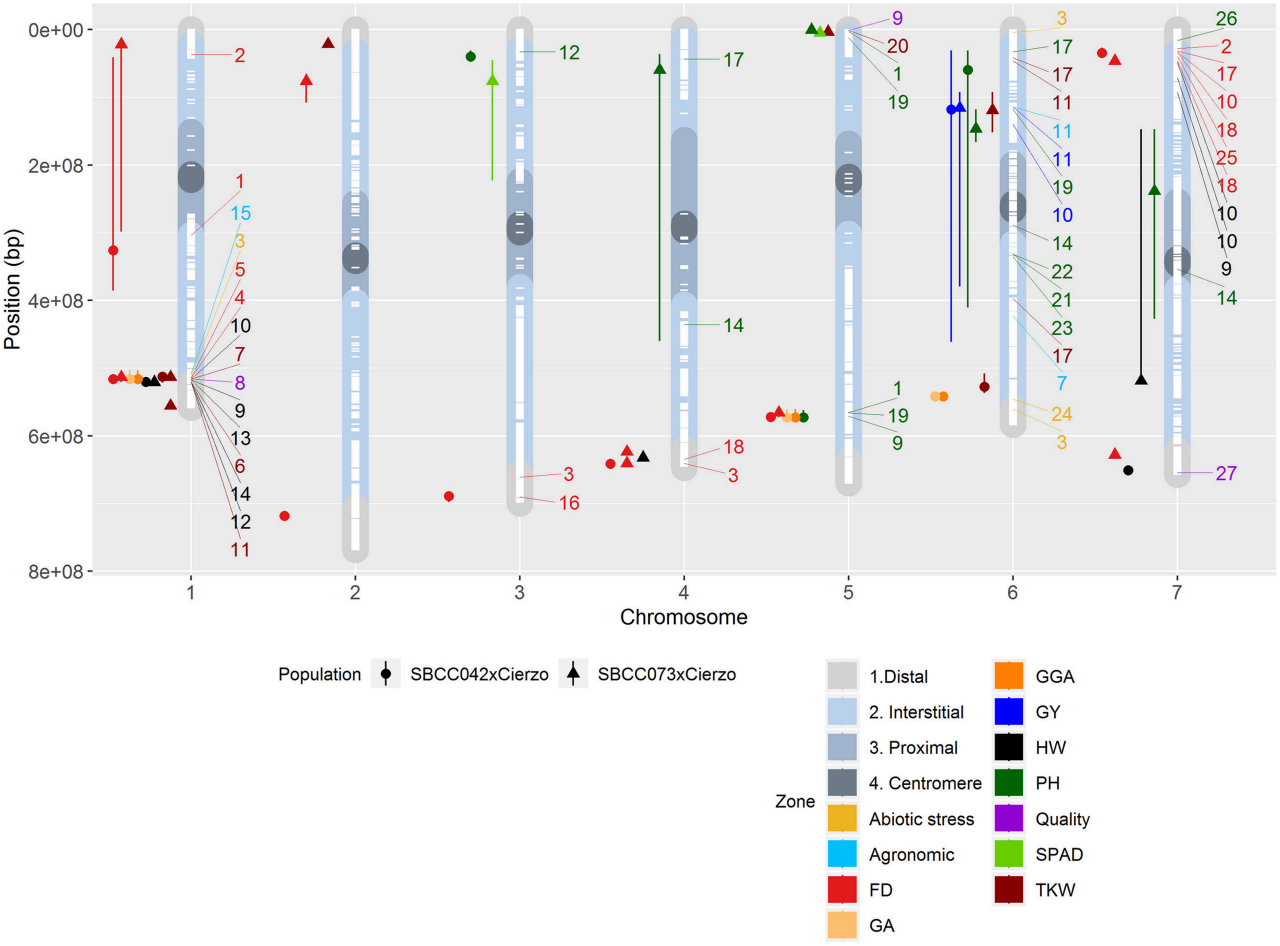
Physical positions of the QTLs identified in the two populations on the seven chromosomes (X-axis): circles, QTLs in SBCC042 × Cierzo; triangles, QTLs in SBCC073 × Cierzo. Chromosome regions represented in color shades: distal in light gray, interstitial in light blue, proximal in gray, and centromere in dark gray. White dashes represent the presence of polymorphic markers in both populations. Colored icons to the left of each chromosome represent the location of a QTL, with whiskers indicating the confidence intervals. FD, flowering date; GA, green area February 2016; GGA, greener area February 2016; HW, hectoliter weight; PH, plant height; TKW, thousand-kernel weight; GY, yield. Colored numbers to the right of each chromosome represent the location of a QTL in the literature: 1, Wonneberger et al. (2017); 2, Alqudah et al. (2014); 3, Rollins et al. (2013); 4, Cuesta-Marcos et al. (2008); 5, Tondelli et al. (2014); 6, Haseneyer et al. (2010); 7, Locatelli et al. (2013); 8, Hassan et al. (2017); 9, Pauli et al. (2014); 10, Nice et al. (2017); 11, Sharma et al. (2018); 12, Rode et al. (2012); 13, Wang et al. (2012); 14, Mansour et al. (2014); 15, Schmalenbach et al. (2011); 16, Lakew et al. (2013); 17, Maurer et al. (2016); 18, Fisk et al. (2013); 19, Alqudah et al. (2016); 20, Mohammadi et al. (2015); 21, Teulat et al. (2001); 22, von Korff et al. (2008); 23, Baum et al. (2003); 24, Ingvordsen et al. (2015); 25, Maurer et al. (2015); 26, Al-Abdallat et al. (2017); 27, Houston et al. (2014). QTLs in the literature whose traits were related to those in our study are designated with the reference number and a color code according to a general trait classification: agronomic represents threshability, grain width and spikes m^−2^; abiotic stress denotes aboveground mass and growth vigor; and quality represents arabinoxylan content, grain plumpness and ß-glucan content.

We detected 21 significant QTLs in SBCC042 × Cierzo and 23 in SBCC073 × Cierzo (Tables 3, 4). Trait-increasing alleles for GY (Figure 2) were only contributed by the elite parent, whereas PH (Figure 2) and TKW (Figure S13) were mainly, but not only, contributed by the landraces. There were many FD QTLs (Figure 2), with trait-increasing alleles contributed by the two parents in both crosses. Ground cover QTL traits were detected only in SBCC042 × Cierzo, with trait-increasing alleles contributed mainly by the landrace (Figure S14). The QTL confidence intervals were cross-referenced to the barley reference sequence and common markers were projected based on other published studies when possible (Figure 3). We identified 5 chromosomal regions with QTLs for one or more phenotypic traits shared across populations and/or years (Figures 3, S15, S16):

- The region at ~35 cM on 1H presented an FD QTL × E with large physical confidence intervals in both populations, which were partially overlapping (QFD.73 × C.1.1 and QFD.42 × C.1.1) and covered almost all of the short arm and the centromeric region. In both cases, the landrace allele contributed earliness, although only in 2015. However, both QTLs corresponded to different physical positions in the reference genome (41–385 and 18–298 Mb, respectively).
- The region at ~75 cM on 1H, one of the aforementioned QTL hotspots, presented significant QTLs with confidence intervals from 503 to 526 Mb. There were QTLs for FD, HW and TKW in both populations, and GA and GGA in SBCC042 × Cierzo. In both populations, flowering was accelerated by the landrace allele, which also supported a higher TKW, and better ground cover was contributed only by SBCC042. The highest HW peak of both populations was at this hotspot at 1H, with a strong trait-decreasing effect of both landrace alleles.
- For the region at ~640 Mb in 4H, both populations presented a QTL for FD, although with contrasting allelic effects across years for SBCC042 × Cierzo.
- The region at ~88 cM for SBCC042 × Cierzo and 132 cM in SBCC073 × Cierzo on 5H (confidence intervals between 562 and 577 Mb) presented an FD QTL in both populations and a QTL for PH and GA in only the former.
- For the region on 6H, at ~19 cM in linkage group 6b SBCC042 × Cierzo and 55 cM in SBCC073 × Cierzo, the QTL confidence intervals were quite large, extending up to 31 to 461 Mb, i.e., covering ~70% of the physical length of the chromosome. Several QTLs with overlapping confidence intervals indicated a higher GY and a lower PH and TKW (only for SBCC042 × Cierzo) obtained from the elite allele.

**Table 3:** Position of peaks and confidence intervals for all QTLs found in the two barley BC_2_F_5_ populations in 2015 and 2016.

**QTL ID**	**Closest marker**	**Linkage** **group**	**Pos. (cM)**	**–log10 (*P*)**	**QTL × E**	**Confidence** **interval (cM)**	**Physical position** **(bp)**	**Confidence interval** **(bp)^#^**
QFD.73 × C.1.1	JHI-Hv50k-2016-15964	1	32.40	4.1	Yes	25.67–39.61	21796427	18775643–298475345
QFD.42 × C.1.1	BOPA2_12_31381	1	38.32	9.1	Yes	34.38–41.73	325808056	41084800–385659752
QTKW.42 × C.1.1	JHI-Hv50k-2016-41900	1	73.67	10.3	–	72.15–78.46	512560611	509509788–521565862
QFD.73 × C.1.2	JHI-Hv50k-2016-42385	1	73.90	4.0	No	73.48–78.61	513317848	511812260–520641451
QTKW.73 × C.1.1	JHI-Hv50k-2016-42385	1	73.9	4.0	–	73.48–78.22	513317848	511812260–519000068
QFD.42 × C.1.2	JHI-Hv50k-2016-43370	1	75.55	25.8	No	74.93–75.87	516310175	515500438–516478745
QGAF.42 × C.1.1	JHI-Hv50k-2016-43370	1	75.55	4.8	–	68.78–82.35	516310175	503268779–522789133
QGGAF.42 × C.1.1	JHI-Hv50k-2016-43370	1	75.55	5.1	–	69.08–82.35	516310175	503501202–522789133
QHW.42 × C.1.1	JHI-Hv50k-2016-45075	1	78.14	37.9	–	77.17–78.46	520099770	519648766–521565862
QHW.73 × C.1.1	JHI-Hv50k-2016-45283	1	79.00	42.9	–	78.82–83.9	520671153	518769706–526169254
QTKW.73 × C.1.2	BOPA1_4057-2114	1	116.00	4.2		110.58–116.6	555618219	550242008–558418541
QTKW.73 × C.2.3	SCRI_RS_153226	2	25.80	4.1		18.71–29.28	21536161	14252491–24793221
QFD.42 × C.2b.3	JHI-Hv50k-2016-125740	2b	53.46	12.6	No	51.99–58.57	718211000	715579692–724202426
QFD.73 × C.2.3	JHI-Hv50k-2016-81375	2	64.50	5.4	No	63.11–67.77	75980517	66004942–107638685
QSPAD.73 × C.3.1	BOPA1_6354-1193	3	45.30	3.6	–	33.83–53.85	76088289	45323182–222788319
QPH.42 × C.3.1	BOPA2_12_10114	3	51.80	4.8	Yes	51.22–52.36	39504125	30927569–44583033
QFD.42 × C.3.4	JHI-Hv50k-2016-223083	3	154.14	5.6	No	150.28–160.46	688991999	682762959–697989230
QPH.73 × C.4.1	JHI-Hv50k-2016-236916	4	49.40	5.9	No	46.5–52.79	59721743	36248096–459813388
QFD.73 × C.4.4	JHI-Hv50k-2016-267120	4	100.30	10.4	No	99.89–101.02	623512998	622953249–626842826
QHW.73 × C.4.2	JHI-Hv50k-2016-269631	4	111.30	4.5	–	102.15–114.36	632281333	626928609–635783191
QFD.73 × C.4.5	JHI-Hv50k-2016-273111	4	122.90	5.9	No	120.47–126.77	640644527	639392943–641279735
QFD.42 × C.4.5	JHI-Hv50k-2016-273856	4	154.05	5.2	Yes	144.02–159.67	641157956	639215315–645128458
QPH.73 × C.5.2	JHI-Hv50k-2016-276967	5	0.00	6.3	No	0–7.4	252867	252867–4137585
QTKW.73 × C.5.4	JHI-Hv50k-2016-278100	5	3.40	6.5	–	0–7.4	3440904	252867–4137585
QSPAD.73 × C.5.2	SCRI_RS_228061	5	12.20	3.8	–	7.4–19.18	4734182	4125224–6667855
QFD.42 × C.5.6	JHI-Hv50k-2016-322900	5	88.14	11.6	No	86.75–93.06	572153706	569271794–577139734
QPH.42 × C.5.2	JHI-Hv50k-2016-323423	5	89.52	5.7	No	83.52–92.17	572441244	562050358–575311094
QGAF.42 × C.5.2	JHI-Hv50k-2016-323423	5	89.52	3.7	–	82.24–90.68	572441244	561003135–572734351
QGGAF.42 × C.5.2	JHI-Hv50k-2016-323423	5	89.52	3.6	–	82.24–90.68	572441244	561003135–572734351
QFD.73 × C.5.6	JHI-Hv50k-2016-321736	5	131.90	5.3	No	127.46–137.87	565947270	562553277–572546090
QPH.42 × C.6b.3	BOPA2_12_30133	6b	18.45	11.8	No	13.59–23.11	59558362	31017496–410500947
QGY.42 × C.6b.1	JHI-Hv50k-2016-390791	6b	19.79	6.8	Yes	13.59–26.27	117897048	31017496–461079345
QTKW.42 × C.6b.2	BOPA1_4146-1154	6b	31.87	7.1	–	30.72–35.39	527210221	507761282–536786820
QGAF.42 × C.6b.3	JHI-Hv50k-2016-417204	6b	43.03	3.7	–	36.32–48.67	541691579	537678975–546597573
QGGAF.42 × C.6b.3	JHI-Hv50k-2016-417204	6b	43.03	3.7	–	35.39–48.67	541691579	536596609–546597573
QGY.73 × C.6.1	SCRI_RS_221874	6	53.70	7.7	No	52.62–57.54	115815022	92140639–379349200
QTKW.73 × C.6.5	SCRI_RS_136897	6	54.30	13.4	–	52.62–54.55	119005613	92140639–151880061
QPH.73 × C.6.3	SCRI_RS_156115	6	54.60	27.8	No	54.34–55.64	146371250	117897048–166244069
QFD.42 × C.7.7	JHI-Hv50k-2016-458320	7	54.16	7.0	No	47.97–55.14	34398097	26408386–37126051
QFD.73 × C.7.7	JHI-Hv50k-2016-461193	7	37.60	6.5	Yes	34.29–39.61	46393227	41833532–47038567
QPH.73 × C.7.4	SCRI_RS_148407	7	67.90	5.0	No	65.72–68.48	238445817	147026927–427236092
QHW.73 × C.7.3	JHI-Hv50k-2016-489580	7	74.60	4.6	–	65.72–75.25	518602866	147026927–521711095
QFD.73 × C.7.8	JHI-Hv50k-2016-505356	7	138.60	6.0	No	137.26–144.95	627972394	627502501–631019235
QHW.42 × C.7.2	JHI-Hv50k-2016-517044	7	156.10	4.2	–	147.22–158.95	651009052	649693674–652764553

*QTLs are coded as “Q”plus the population (42 × C or 73 × C), followed by the chromosome number and sequential number of QTLs for the trait and population. When applicable, whether the QTL was detected as a main effect or as a QTL by environment interaction (QTL × E) it is indicated. Confidence intervals are given in genetic and physical positions*.

# *Confidence interval in the physical map (Mascher et al., 2017) based on Bayes credible intervals*.

**Table 4:** Percentage of variance explained for and additive effect (effect of replacing an allele from parent 1, Cierzo, with an allele from parent 2, landrace) of the QTLs found in the two barley BC_2_F_5_ populations in 2015 and 2016.

	**% Of variance explained**		**Additive effect**	
**QTL ID**	**2015**	**2016**	**2015**	**2016**
QFD.73 × C.1.1	8.4	0.0	0.650^***^	−0.021^ns^
QFD.42 × C.1.1	11.3	0.1	0.878^***^	0.084^ns^
QTKW.42 × C.1.1		22.5	−1.503	
QFD.73 × C.1.2	8.0	10.7	0.637^***^	0.637^***^
QTKW.73 × C.1.1		12.0	−1.059	
QFD.42 × C.1.2	24.7	27.5	1.297^***^	1.297^***^
QGAF.42 × C.1.1		11.1	−0.022	
QGGAF.42 × C.1.1		11.9	−0.020	
QHW.42 × C.1.1		72.8	2.482	
QHW.73 × C.1.1		167.4	3.065	
QTKW.73 × C.1.2		6.9	0.802	
QTKW.73 × C.2.3		7.8	−0.854	
QFD.42 × C.2b.3	16.0	17.8	1.043^***^	1.043^***^
QFD.73 × C.2.3	9.5	12.7	−0.692^***^	−0.692^***^
QSPAD.73 × C.3.1		42.9	0.601	
QPH.42 × C.3.1	30.9	4.4	4.197^***^	1.2466^ns^
QFD.42 × C.3.4	7.7	8.6	−0.727^***^	−0.727^***^
QPH.73 × C.4.1	10.2	12.6	2.325^***^	2.325^***^
QFD.73 × C.4.4	19.1	25.5	0.982^***^	0.982^***^
QHW.73 × C.4.2		9.8	−0.742	
QFD.73 × C.4.5	18.1	24.2	−0.957^***^	−0.957^***^
QFD.42 × C.4.5	3.1	2.8	0.461^**^	−0.411^*^
QPH.73 × C.5.2	6.3	7.8	−1.827^***^	−1.827^***^
QTKW.73 × C.5.4		16.2	−1.230	
QSPAD.73 × C.5.2		10.9	−0.291	
QFD.42 × C.5.6	13.5	15.1	−0.960^***^	−0.960^***^
QPH.42 × C.5.2	7.5	12.3	−2.073^***^	−2.073^***^
QGAF.42 × C.5.2		11.0	−0.022	
QGGAF.42 × C.5.2		10.5	−0.019	
QFD.73 × C.5.6	9.6	12.9	−0.698^***^	−0.698^***^
QPH.42 × C.6b.3	15.8	25.7	−2.996^***^	−2.996^***^
QGY.42 × C.6b.1	17.3	3.7	0.443^***^	0.178^*^
QTKW.42 × C.6b.2		15.7	−1.257	
QGAF.42 × C.6b.3		9.3	−0.021	
QGGAF.42 × C.6b.3		9.2	−0.018	
QGY.73 × C.6.1	12.6	6.4	0.232^***^	0.232^***^
QTKW.73 × C.6.5		22.7	−1.458	
QPH.73 × C.6.3	24.1	29.9	−3.574^***^	−3.574^***^
QFD.42 × C.7.7	5.7	6.4	0.625^***^	0.625^***^
QFD.73 × C.7.7	16.1	2.9	−0.902^***^	−0.333^*^
QPH.73 × C.7.4	4.2	5.2	−1.491^***^	−1.491^***^
QHW.73 × C.7.3		6.3	0.593	
QFD.73 × C.7.8	9.7	13.0	0.701^***^	0.701^***^
QHW.42 × C.7.2		11.8	1.000	

*, **, *** *Significant effect at P < 0.05, P < 0.01, and P < 0.05, respectively (calculated only for traits measured in two trials); ns, non-significant effect*.

In addition to these QTLs, we found an FD QTL on the short arm of 7H in both populations. However, only the QTL in SBCC073 × Cierzo seemed to be near the well-known *HvFT1* gene. Flowering was accelerated by the elite cultivar allele in both populations. For early vigor (measured as GA and GGA), only SBCC042 contributed trait-increasing alleles (GA_Feb and GGA_Feb in 2016) with three significant QTLs (1H, 75.5 cM; 5H, 89.5 cM; and 6Hb, 43.0 cM). These findings indicate an increase in ground coverage after winter, which might be exploited as an adaptive mechanism of this Spanish landrace.

Interactions between QTLs were tested for all possible pairs of markers representing QTL peaks for each trait. We did not expect to find highly significant correlations, as this kind of population presents such imbalanced genotypic frequencies. We considered interactions that had a minimum number of 5 lines in each of the four possible genotypic classes (considering only homozygotes at each pair of genes). There were two significant interactions, one for hectoliter weight, i.e., QHW.73 × C.1.1 × QHW.73 × C.4.2 (*P* = 0.030, Figure S17a), and one for plant height, i.e., QPH.73 × C.4.1 × QPH.73 × C.7.4 (*P* = 0.011, Figure S17b).

### Validation of SBCC073 × Cierzo QTLs

The field trial carried out with 96 lines of this population confirmed the GY QTL, 3 of the 4 PH QTLs and 3 of the 5 TKW QTLs. None of the FD QTLs were confirmed. This result was expected, as this subpopulation was chosen based on its homogeneous FD, and the extremes of the FD distribution were discarded. The estimation of yield components and the harvest index allowed investigation of the causes underlying the grain yield QTL found on 6H. Biomass production did not differ between the alleles (83.72 vs. 83.84 g) or the number of tillers and spikes per unit area. Differences in GY resulted from a large divergence in the harvest index, which, was in turn mainly caused by the significantly larger number of grains (14%) exhibited by lines with the Cierzo allele (Table 5).

**Table 5:** Results of the validation trial for the marker at the QGY.73 × C.6.1 peak in the 2017–18 season.

**Trait**	**SCRI_RS_221874** **Cierzo allele**	**SCRI_RS_221874** **SBCC073 allele**
*n* (number of lines)	66	15
Grain yield (k·ha^−1^)	2918	2619^**^
Thousand kernel weight (g)	29.4	32.1^**^
Harvest index	52.4	49.2^**^
Grains per spike	29.5	25.5^**^
Number of spikes per sample	51.4	51.0^ns^
Heading date (Julian date)	117.8	118.7^**^
Plant height (cm)	71.1	76.2^**^
Shoot biomass (g sample^−1^)	83.7	83.8^ns^

** *significant at P < 0.01; ns, non-significant*.

## Discussion

### Can We Identify Candidate Loci Underlying QTL Using the 50k Chip and Two Large Populations?

Identifying candidate genes with the sizes and types of populations and density of markers used here is possible only for loci with very large phenotypic effects and little genotype-by-environment interaction, and we did not find any such loci. However, there is a lot to gain from the use of the 50k chip and the current reference barley genome (Mascher et al., 2017) from the point of view of barley breeding. Good marker coverage combined with a large population size narrowed down the confidence intervals for many QTLs, reducing the list of potential candidates (Supplementary Data 2). These shortlists are trustable resources for further research because there is high confidence in the physical position of the genes. QTL flanking markers from the literature were cross-referenced with ours, as the use of a reference genome helps to confirm or reject commonalities among findings. In some cases, we were able to shed new light on candidates proposed in previous studies.

The use of the reference genome has some small caveats, such as the presence of unexpected duplications in the genome, which complicate map construction. For instance, locus *HvFT1* appears on both chromosomes 3H (HORVU3Hr1G087100) and 7H (HORVU7Hr1G024610). We placed *HvFT1* only on 7H in our maps based on a linkage map (BOPA2_12_30893) and the abundant literature that reports the locus being located on this chromosome instead of 3H. Some duplications may be real, but geneticists should be aware of this fact and apply their expertise and previous knowledge in each case.

Below, we report insights on the QTLs found, i.e., matches with previous studies, and information on genes of interest in the QTL regions in chromosome order. We do not intend to declare candidate genes. Rather, we combine the information with possible biological meaning that we found after cross-referencing functional and positional information, aiming to pose meaningful questions:

The flowering date QTLs found at ~30–40 cM in chromosome 1H on both populations (QFD.73 × C.1.1 and QFD.42 × C.1.1) have overlapping confidence intervals. However, the intervals are large, and the peaks fall on different arms for the two populations (1HS and 1HL, respectively). The peak marker for the first QTL falls within a gene annotated as *MYB DOMAIN PROTEIN 87* and is close to a flowering date QTL described in Wonneberger et al. (2017). For SBCC073 × Cierzo, the interval contains a *CYTOCHROME P450 SUPERFAMILY PROTEIN* (*HORVU1Hr1G009110*), *HORVU1Hr1G010780* (*SENSITIVITY TO RED LIGHT REDUCED PROTEIN, SRR1*), which is polymorphic in both populations, and *HORVU1Hr1G011800*, which is *HvTOE1*, an ortholog of *TaTOE-B1* (Zikhali et al., 2017). This last gene is an *AP2-LIKE ETHYLENE-RESPONSIVE TRANSCRIPTION FACTOR*, located at 36.02 cM, coincident with a QTL for time to awn tipping reported by Alqudah et al. (2014) that was polymorphic only in the second population. TOE proteins in *Arabidopsis* “convey a photoperiodic signal to antagonize CONSTANS and regulate flowering time” (Zhang et al., 2015).

The QTL hotspot at 503–526 Mb on 1H can actually be split into two spots based on the overlap of confidence intervals and the underlying genes. The first spot, to the left of the region, harbors the QTLs for FD and TKW in both populations and the GA QTL in SBCC042 × Cierzo. The second spot, to the right, encompasses the HW QTL for both populations. The QTLs for FD and TKW are located between 509 and 521 Mb. Both landraces have an active *HvFT3* allele, which Cierzo lacks. This gene has a large effect on FD in Mediterranean conditions (Boyd et al., 2003; Cuesta-Marcos et al., 2008; Tondelli et al., 2014) and could be responsible for the detected QTLs with earlier landrace alleles. However, the confidence interval in SBCC042 × Cierzo is slightly shifted to the right of *HvFT3*. Another gene with an effect on FD in plants is *HORVU1Hr1G076800* (*DOF ZINC FINGER PROTEIN 2*), located at 515.7 Mb, 75 cM in this population. Genes of this family of transcription factors repress *CONSTANS* in *Arabidopsis*, delaying flowering (Fornara et al., 2009). The effect on TKW (increased by landrace alleles) could be pleiotropic or could be due to another gene. Other studies revealed QTLs for TKW (Haseneyer et al., 2010; Locatelli et al., 2013) and arabinoxylan content (Hassan et al., 2017) in this same region. Neither of the studies reported any association with earliness; thus, the authors discarded *eam8* (*ELF3*, Faure et al., 2012; Zakhrabekova et al., 2012) as a candidate. Pauli et al. (2014) found a novel QTL for test weight in this region, which they reported as different from the QTL in the region of *PpdH2*. However, the projection of their confidence interval onto the physical map indicates that the QTL found by these authors corresponds to the region we identified near *HvFT3*. Even in such a small region, several genes could be candidates for this effect. The genes *HEXOKINASE 1* and *TREHALOSE PHOSPHATE SYNTHASE* are located in the confidence intervals (511 and 514 Mb) and were found to play a role in repressing and/or redirecting sucrose utilization in barley caryopses during heat stress exposure (Mangelsen et al., 2011). In fact, markers for this last gene are polymorphic in both populations.

The bimodal distribution of phenotypic frequencies for HW (Figure S3) hints at the possibility of a major gene segregating for this trait in both populations for QHW.42 × C.1.1 and QHW.73 × C.1.1. These QTLs, between 519 and 526 Mb, present larger values for the Cierzo allele in both populations. Studies crossing wild and cultivated barley have found QTLs in the same region. Nice et al. (2017) found an HW (also named test weight) QTL, and Sharma et al. (2018) found a TKW QTL, with the wild allele contributing lower values. Studies with cultivated barley also found QTLs for HW in the same area (Rode et al., 2012; Wang et al., 2012; Mansour et al., 2014). Interestingly, all these QTLs collocate with the threshability locus *thresh-1*, identified in another wild-by-cultivated cross (Schmalenbach et al., 2011), although we can discard the candidates they proposed because the candidates fall outside our confidence intervals.

QFD.73 × C.2.3 lies just 7 Mb from *HvFT4*, although this gene is not represented in the 50k chip. Two more genes of the *CYTOCHROME P450 SUPERFAMILY* (*HORVU2Hr1G025160* and *HORVU2Hr1G025480*) are inside the confidence interval. Farther down chromosome 2H, the QFD.42 × C.2b.3 peak is just 1 Mb away from *HvARF9*, an *AUXIN RESPONSE FACTOR*.

At QPH.42 × C.3.1, the Cierzo allele contributes increased plant height. QTL for plant height in this region were previously reported by Haseneyer et al. (2010) and by Rode et al. (2012), although marker comparison confirmed the colocation of QTLs for only the second QTL.

QFD.42 × C.3.4 falls in the same region as a flowering time QTL found in the population Arta × Keel (Rollins et al., 2013), in which the Arta allele conferred lateness, as did SBCC042. Interestingly, Arta is a Mediterranean landrace from the Middle East. Lakew et al. (2013) also detected a flowering time QTL in the same region. We can discard the *denso*/*sdw1* region, at ~630 Mb, as responsible for our QTL. Our confidence interval (682–698 Mb) actually includes the region of the earliness gene *eam10* (*HvLUX*, 692 Mb, Campoli et al., 2013).

At the plant height QTL QPH.73 × C.4.1, the Cierzo allele also increases plant height. Maurer et al. (2016) found a PH QTL, with a wild barley allele contributing increased height, and Mansour et al. (2014) found another PH QTL in the population between Cierzo parents. In the QTL peak we found an *ACETYL ESTERASE*, corresponding to a gibberellin (GA) receptor (GID1L2-8, GA-Insensitive Dwarf 1, Hill et al., 2018).

The possibly common FD QTL found at both populations on 4H is located close to *VrnH2*. However, all parents have an active *VrnH2* allele (all three *ZCCT-H* genes); therefore, it is unlikely to be the causal gene. Fisk et al. (2013) and Rollins et al. (2013) found an FD QTL in this location, but in both cases, they were consistent with a *VrnH2* effect. However, a possible causal effect of *VrnH2* cannot be ruled out without further evidence. Another gene present in the vicinity of this QTL is *HORVU4Hr1G088850*, which codes for a *PROTEIN CHAPERONE-LIKE PROTEIN OF POR1*, that is essential for chloroplast development (Lee et al., 2013).

The peak of QPH.73 × C.5.2 falls exactly on *HORVU5Hr1G0000010, SUCROSE TRANSPORTER 4*. Wonneberger et al. (2017) reported a PH QTL in the same region.

QTKW.73 × C.5.4 (with SBCC073 contributing a larger grain weight) is in the same region as QTLs reported by Pauli et al. (2014) for grain plumpness, and by Mohammadi et al. (2015) for TKW.

The QTL hotspot on 5H between 561 and 577 Mb holds QTLs for FD in both populations and for PH and GA in SBCC042 × Cierzo, in all cases with landrace trait-increasing alleles. It is interesting that *TWO-COMPONENT RESPONSE-REGULATOR-LIKE PRR95*, a circadian clock gene (Campoli et al., 2012; Calixto et al., 2015) is close to the peak for the FD QTL. Although the gene is located slightly outside the confidence interval in SBCC042 × Cierzo and thus should not be highlighted as a possible candidate, we found some inconsistencies between our maps and the reference genome in the region and thus cannot discard *HvPRR95*.

In SBCC042 × Cierzo, the landrace allele also contributes a larger GA, which could be related to the more vigorous early shoot growth exhibited by SBCC042 compared to that in either Cierzo or SBCC073 (Table 1). Ceccarelli et al. (1991) reported that early vigor leads to adaptation to marginal environments. Early vigor could also be related to differential responses to frost damage, although we think this relationship is unlikely in our case, as no frost damage was detected in the trials.

From an agronomic point of view, the most important QTL hotspot found in our study is the one on 6H. As mentioned before, this region is very wide. It spans both chromosome arms, although all QTL peaks lie on the short arm. The marker on the peak for QPH.42 × C.6b.3 cosegregates with *HORVU6Hr1G020330*, which corresponds to *AUXIN RESPONSE FACTOR 19* (Tombuloglu, 2018), and there is another auxin response gene very close to the peak in the first population, *HORVU6Hr1G021040*, which corresponds to *AUXIN SIGNALING F-BOX 3*. The finding of this QTL in both populations could be due to the excessive plant height of landraces used for modern breeding (Yahiaoui et al., 2014). Mansour et al. (2014) also found a QTL for PH in the same region (325 Mb) in the population Orria × Plaisant, with the Orria allele reducing plant height. Other studies have found PH QTLs on 6H with overlapping regions in very different germplasm sets, such as Alqudah et al. (2016) in a GWAS study, Maurer et al. (2016) in a wild-by-cultivated cross, and Teulat et al. (2001) and von Korff et al. (2008) in Mediterranean barleys. Baum et al. (2003) also found a PH QTL on 6H. It is not possible to conclude that all these studies identified one or several underlying genes, but our findings on this 6H region confirm its key role in barley breeding.

Regarding grain yield and yield components at the 6H hotspot, the QTL for GY in both populations and the TKW QTL on SBCC073 × Cierzo show close peak positions (115–119 Mb), suggesting a unique QTL. Sharma et al. (2018) and Nice et al. (2017) also found GY QTLs in the vicinity (114 Mb and 140 Mb, respectively), with the yield-decreasing allele contributed by wild parents. In contrast, Locatelli et al. (2013) found a QTL for spikes per square meter (423 Mb). The validation trial suggested a major role of the harvest index, through both grain number per spike and TKW. Inside the confidence intervals and very close to the GY peaks (114–117 Mb), there are several interesting genes that are polymorphic in both populations: *HORVU6Hr1G029150* (*UBIQUITIN CARBOXYL-TERMINAL HYDROLASE 2*), which affects shoot architecture in *Arabidopsis* (Yang et al., 2007); *HORVU6Hr1G029160* (*PROTEIN PHOSPHATASE 2C FAMILY PROTEIN*), a key player in signal transduction (Rodriguez, 1998), which is polymorphic in the first population; *HORVU6Hr1G028680*, a *TWO-COMPONENT RESPONSE REGULATOR ARR12* (Moubayidin et al., 2010); and *HORVU6Hr1G028710*, a *GLUCOSE-6-PHOSPHATE DEHYDROGENASE 2*. A locus with a large effect on grain weight on chromosome 6 has been detected in syntenic regions of other cereals. *OsGW2* in rice (Song et al., 2007) and its homolog in wheat, *TaGW2* in the A genome are associated with TKW (Su et al., 2011) and grain yield (Simmonds et al., 2014). However, its apparent homolog in barley, *HORVU6Hr1G044080*, annotated as *PROTEIN SIP5* (251 Mb), was included in our GY QTL confidence intervals but was far from the peaks and not located in the TKW one. It seems unlikely that the QTLs detected in our study are caused by the barley *GW2* homolog.

There was another TKW QTL on 6H, but only in SBCC073 × Cierzo. The peak falls on the gene *HORVU6Hr1G076810, PYRUVATE DEHYDROGENASE E1 COMPONENT SUBUNIT ALPHA*, and also includes the gene *HORVU6Hr1G076760, ARM REPEAT SUPERFAMILY PROTEIN* (Mudgil et al., 2004). Based on position, we can discard coincidence with *HvNAM-1* (grain protein content), *HORVU6Hr1G019380* (Distelfeld et al., 2008), which is associated with variation in TKW in population HEB-25 (Maurer et al., 2016).

Finally, on 6H, there is a GA QTL in SBCC042 × Cierzo. GA/GGA in SBCC042 × Cierzo is associated with increased green area conferred by the SBCC042 allele. Rollins et al. (2013) found a growth vigor QTL in Arta × Keel, with a trait-increasing allele from the landrace Arta, whose interval did not encompass ours. Ingvordsen et al. (2015) also found a nearby QTL for aboveground biomass. The gene *HORVU6Hr1G080340, ETHYLENE-RESPONSIVE TRANSCRIPTION FACTOR 5*, is located within our confidence interval.

The two FD QTLs found on the short arm of 7H occur at close but non-overlapping positions. The well-known gene *HvFT1*, which has a large effect on FD, falls between the two QTLs (40 Mb) and outside their confidence intervals and therefore cannot be considered a candidate. In both populations, Cierzo contributes the early allele. Coincident with QFD.42 × C.7.7, Nice et al. (2017) reported an FD QTL in an AB-NAM population (*HORVU7Hr1G022550*, at 33 Mb). Fisk et al. (2013) and Alqudah et al. (2014) also found several QTLs related to flowering time or phasal growth duration coincident with this region but, based on the location of their associated markers on the current reference genome, they seem distal to *HvFT1*. The gene *HORVU7Hr1G026840, HISTONE-LYSINE N-METHYLTRANSFERASE E(Z)*, which participates in histone methylation is located very close to the QFD.73 × C.7.7 peak. An FD QTL in the HEB-25 population, with the later allele coming from the wild barleys, was reported in the same region (Maurer et al., 2015, 2016), and another FD QTL in cultivated barleys was reported by Fisk et al. (2013). The fact that we found two FD QTLs on the short arm of 7H that were close to but apparently different from *HvFT1* questions the nature of the genes underlying flowering time QTLs found in a large number of studies in this region.

QPH.73 × C.7.4 and QHW.73 × C.7.3 show distant peaks, but their wide confidence intervals greatly overlap. The landrace allele increases plant height and reduces hectoliter weight. Regarding HW, the test weight QTL found by Nice et al. (2017) is located outside our confidence interval. Previously, PH QTLs were found in Orria × Plaisant (Mansour et al., 2014), and in a GWAS study with Jordanian landraces (Al-Abdallat et al., 2017). The first one falls within our confidence interval. At the peak for PH, we found the genes *HORVU7Hr1G056590* (*HYDROXYMETHYLGLUTARYL-COA SYNTHASE*), *HORVU7Hr1G056630* (*RHOMBOID-LIKE PROTEIN 3*), *HORVU7Hr1G056980* (*6-PHOSPHOGLUCONOLACTONASE 5*) and *HORVU7Hr1G057100* (*CYTOCHROME P450 SUPERFAMILY PROTEIN*), which could be related to the trait. In addition, two genes are present in the region of the QTL peak, namely, *BASIC LEUCINE ZIPPER (bZIP) TRANSCRIPTION FACTOR FAMILY PROTEIN HvIRO1* (AB199587.1 *HORVU7Hr1G056490*) and the gibberellin receptor *GID1* (GA-insensitive dwarf phenotype, *HORVU7Hr1G057260*, not present in the 50k chip). Both are related to the “dwarf” phenotype (Hartweck and Olszewski, 2006; Ogo et al., 2006). The presence of epistasis of this QTL with QPH.73 × C.4.1, which is related to another GA-receptor (GID1L2-8, Hill et al., 2018), leads us to think that the interactions of genes related to the GA pathway are involved in yield-related traits in this population.

At QHW.42 × C.7.2, the Cierzo allele increases HW. The peak falls within the gene *HvBRD2*/*HvDIM, DELTA(24)-STEROL REDUCTASE, HORVU7Hr1G120030*, with putative effect on plant height and tillering formation (Alqudah et al., 2016). The region also coincides with a beta-glucan-content QTL found by Houston et al. (2014).

### Can Spanish Landraces Contribute to Improving Elite Local Cultivars?

Elite cereal cultivars are derived from a relatively narrow germplasm pool and are predominantly well-adapted to high-input agriculture (Newton et al., 2010). This study relies on the assumption that local landraces can contribute adaptive features that may not have been fully incorporated into current elite varieties and that may be particularly relevant as sources of novel “abiotic stress resistance genes or combinations of genes if deployed appropriately” (Newton et al., 2010). In this study, we aimed to detect some genetic factors responsible for the good performance and adaptation of top Spanish barley landraces under Mediterranean conditions, and to transfer them into an elite cultivar.

The two contrasting seasons, in terms of both vegetative development (measured as plant height) and grain yield, provide an opportunity to compare performance under high-yielding conditions (2016) vs. typical stress conditions occurring late in the season (2015).

The elite parent, Cierzo, was used as a yardstick to measure the potential of these two superior landraces to contribute to barley breeding. Unfortunately, we did not detect direct grain yield QTLs with the superior allele coming from the landraces, even in the lower-yielding year. There were QTLs for TKW, HW and FD with trait-increasing alleles from both sides of each cross, indicating that breeding is possible in either direction of these crosses for each of these traits. QTLs for GA (and GGA) were detected only in SBCC042 × Cierzo, although they were moderately and positively correlated with grain yield in both years and in both populations. This finding is consistent with the much higher ground cover shown by SBCC042 than by Cierzo in 2016. Thus, at least under the two contrasting conditions experienced in the two seasons, and for both populations, more profuse development during the vegetative period can be considered a positive feature. This trait is easy to score and to select for, and these results hold promise for further use of this trait in barley breeding. However, the positive correlation between this trait and plant height can be a problem, particularly in SBCC042 × Cierzo, as selection for ground cover could result in indirect selection for taller plants.

The landraces can introduce excessive plant height and low hectoliter weight. We identified some QTLs that could be introgressed together with other desirable alleles to counterbalance these potential negative effects. One of the desirable alleles was the landrace allele at QPH.73 × C.4.1, which reduced plant height. Actually, the height of the elite cultivar Cierzo is acceptable, but is still on the tall side of the range of variation of current cultivars; thus, the cultivar could benefit from height reduction. There are several QTLs with trait-increasing landrace alleles that could be used to increase the kernel weight of Cierzo. Three of the QTLs, namely, QTKW.73 × C.1.1, QTKW.73 × C.4.4, and QTKW.73 × C.6.5, are part of regions with other QTLs with negative contributions from landrace alleles to other agronomic traits, and their use for this purpose is not advisable. However, the landrace allele from QTKW.42 × C.6.2 contributed to a higher kernel weight and was linked to better early ground cover, as it was close to QGA.42 × C.6.3, for which the landrace allele increased early (February) ground cover.

There were 16 and 14 lines in each population with yield values above the elite parent's values plus 2 5% LSDs in each year. These lines will be further tested in field trials, and some may become candidate cultivars.

### Can Spanish Landraces Be Improved to Compete With Current Elite Cultivars?

The development of cultivars, performed since the 1960s, has led to shorter cultivars with faster growth cycles. Spanish barley landraces are generally too tall for current agriculture and are prone to lodging (Yahiaoui et al., 2014). Landrace lines with reduced height would be interesting materials. They could become cultivars directly or at least could be used as parents in plant breeding programs, due to their reduced genetic load. Therefore, plant height reduction is a sensible breeding and pre-breeding target for landrace plant material.

A good number of plant height QTLs were found, with the trait-increasing allele coming from the landrace side, except in one case. There is ample (mostly additive) variation for reducing plant height in these landraces by more than 15 cm, if the effect of all QTLs is considered. However, this trait showed antagonistic associations with kernel weight in our study. Moderate positive correlations (~0.4) between plant height and kernel weight were reported in studies involving Mediterranean germplasm (von Korff et al., 2008; Rollins et al., 2013) under stress conditions. Consequently, this possible association should be taken into account when addressing barley breeding for this kind of environment.

In our populations, the positive PH-TKW correlation (i.e., negative association from an agronomic point of view) occurred in one population for the QTL at the beginning of 5H (QPH.73 × C.5.2 and QTKW.73 × C.5.4) and at the 6H QTL hotspot in both populations, meaning that selection for these regions will affect height and kernel weight in agronomically opposite directions. In the last case (6H), however, it is possible to select for decreased height and increased grain yield at the same time. In both populations, this PH QTL has the largest effect. Therefore, selecting for the elite allele in this single region will combine a large height reduction (6–7 cm, depending on the population) with increased yield. A reduction in kernel weight (2.5–3 g, depending on the population) would still produce lines with acceptable kernels, that are much larger than the elite parent's. Since plant height QTLs on the short arm of 6H are consistently detected in crosses involving germplasm from the Mediterranean region, this approach could be relevant for breeding with other germplasm of Mediterranean origin.

## Author Contributions

EI, AC, and MG conceived this work. MG and EI developed the populations. MG, EI, and AM planned and carried out the field experiments and data collection. AC and AM performed the laboratory work. CC, BC-M, and AM curated and corrected marker data, corrected allele calls. AM performed the QTL analyses, and AM and EI performed other statistical analyses. AM, AC, and EI drafted the document. All the authors read and approved the manuscript.

### Conflict of Interest Statement

The authors declare that the research was conducted in the absence of any commercial or financial relationships that could be construed as a potential conflict of interest.

## Footnotes

^1^http://www.genvce.org/variedades/cebada/invierno/cierzo/ (accessed February 11, 2019).

## Abbreviations

FD: flowering date
GA: green area
GGA: greener area
GY: grain yield
HW: hectoliter (or test) weight
PH: plant height
QTL: quantitative trait locus
SBCC: Spanish Barley Core Collection
SNP, single nucleotide polymorphism. SPAD: chlorophyll content measured with soil plant analysis development (SPAD) chlorophyll meter
TKW: thousand-kernel weight.
