# Supplementary material for: Harnessing Novel Diversity From Landraces to Improve an Elite Barley Variety
Source: Front Plant Sci. 2019 Apr 11;10:434. doi: 10.3389/fpls.2019.00434 (PMC6470277; doi:10.3389/fpls.2019.00434)
Supplement: Supplementary file 1 [file Data_Sheet_1.PDF]

Supplementary Material

Harnessing novel diversity from landraces to improve an elite barley variety

Arantxa Monteagudo, Ana M. Casas, Carlos P. Cantalapiedra, Bruno Contreras-Moreira, M Pilar Gracia, Ernesto Igartua*

*** Correspondence:** Corresponding Author: igartua@eead.csic.es

Supplementary File 3.

Supplementary Tables, S1-S3, and Figures, S1-S17

Table S1. Traits recorded for each population in each field trial.

**Trial**	Grain yield	Plant height	Flowering date	Hectolitre weight	Thousand kernel weight	GA March	GGA March	GA Feb	GGA Feb	SPAD
**SBCC042 x Cierzo 2015**	X	X	X			X	X			
**SBCC073 x Cierzo 2015**	X	X	X			X	X			
**SBCC042 x Cierzo 2016**	X	X	X	X	X			X	X	
**SBCC073 x Cierzo 2016**	X	X	X	X	X			X	X	X

Table S2. Analysis of variance for the agronomic traits measured in two years, for both populations (degrees of freedom, df and mean squares, ms), done with the spatial adjusted values. The F values for “genotype” are approximations, as the residual corresponds to the df for the replicated genotypes, primary checks Cierzo and Orria (28 replicates per year) and secondary checks SBCC073 and Plaisant (8 replicates per year).

	**Yield**				**Plant Height**				**Flowering time**			
	**df**	**ms**	**F. Prob**	**Sign.**	**df**	**ms**	**F. Prob**	**Sign.**	**df**	**ms**	**F. Prob**	**Sign.**
	**-- SBBC042 x Cierzo --**											
**Genotype**	267	1.83	<0.001	*	267	107.13	<0.001	*	267	12.69	<0.001	*
**Year**	1	886.21	<0.001	*	1	26488.01	<0.001	*	1	5276	<0.001	*
Genotype x Year	267	0.81	0.711	ns	267	35.22	0.197	ns	267	2.59	0.355	ns
**Residual**	136	0.88			136	30.91			136	2.45		
**Total**	671	2.55			671	102.38			671	14.44		
	**-- SBBC073 x Cierzo --**											
**Genotype**	267	1.21	<0.001	*	267	95.95	<0.001	*	267	7.29	<0.001	*
**Year**	1	460.51	<0.001	*	1	28418.12	<0.001	*	1	1192.02	<0.001	*
Genotype x Year	267	0.61	0.008	*	267	31.84	0.218	ns	267	2.09	0.381	ns
**Residual**	136	0.42			136	28.26			136	1.99		
**Total**	671	1.50			671	98.93			671	5.92		

* P.value <0.01; ns, not significant;

Table S3. Genetic maps details for both populations, number of markers and distance per linkage group.

Linkage group	Number of markers	Length (cM)	Average of spacing (cM)	Maximum spacing (cM)
**--------------------------------- SBCC042 x Cierzo --------------------------------**				
1	1290	128.5	1.0	9.8
2a	734	94.6	1.6	18.2
2b	1033	96	1.3	11.6
3	2104	160.5	1.0	10.5
4	1611	159.7	1.3	15.4
5	2275	170	1.0	16.8
6a	209	10.6	1.2	3.4
6b	1605	92.7	0.9	12.3
7	2032	168.1	1.1	10.7
**Overall**	**12893**	**1080.6**	**1.1**	**18.2**
**--------------------------------- SBCC073 x Cierzo --------------------------------**				
1	1273	116.6	1.3	8.4
2	1835	174.7	1.1	9.1
3	1862	155	1.6	15.1
4	1419	127.2	1.2	8.6
5	2087	225.4	1.4	12.0
6	1531	139.3	1.3	12.5
7	2019	177.6	1.2	12.9
**Overall**	**12026**	**1115.8**	**1.3**	**15.1**

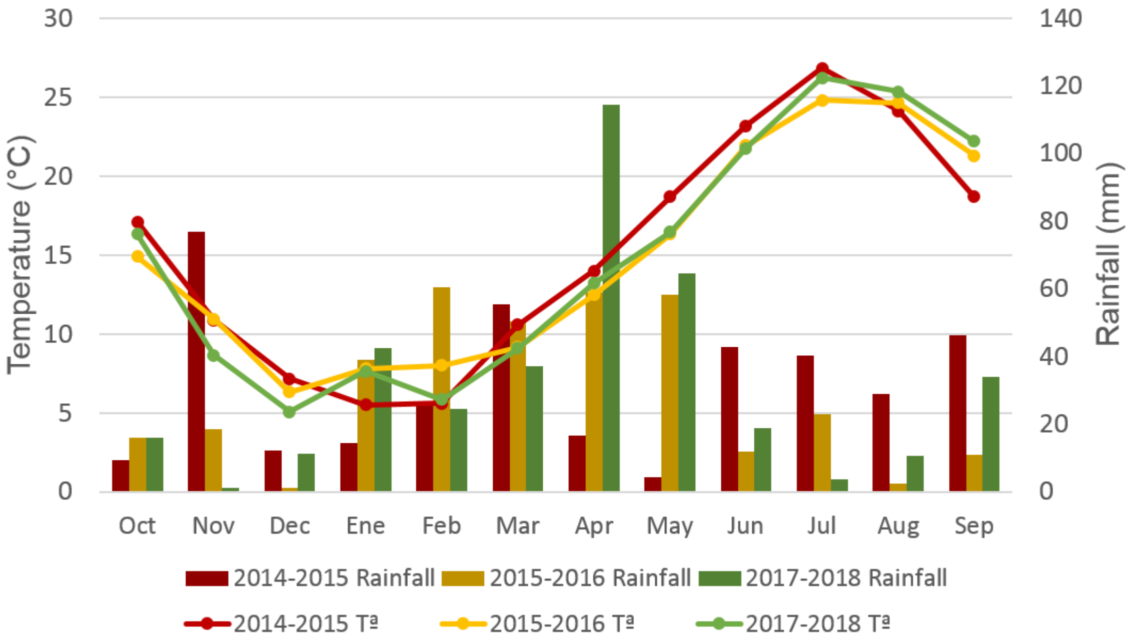

Fig. S1. Climatic conditions in Zuera, in 2014-2015, 2015-2016 and 2017-2018 seasons. Bars represent the accumulated rainfall in each month. Lines indicate the average of temperature in a monthly basis.

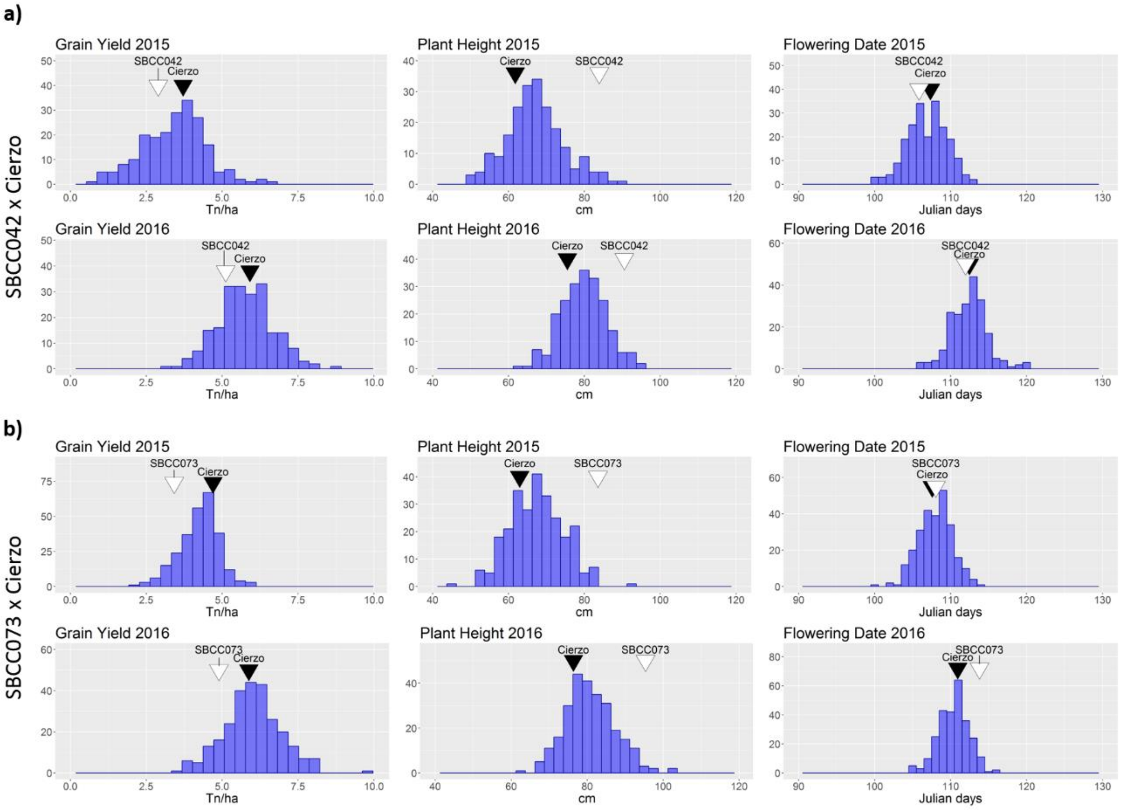

Figure S2. Histogram of distributions of grain yield, plant height and flowering date for the two seasons. a) SBCC042 x Cierzo. b) SBCC073 x Cierzo. Cierzo, SBCC042 and SBCC073 represent an average of 28, 8 and 8 replicates, respectively.

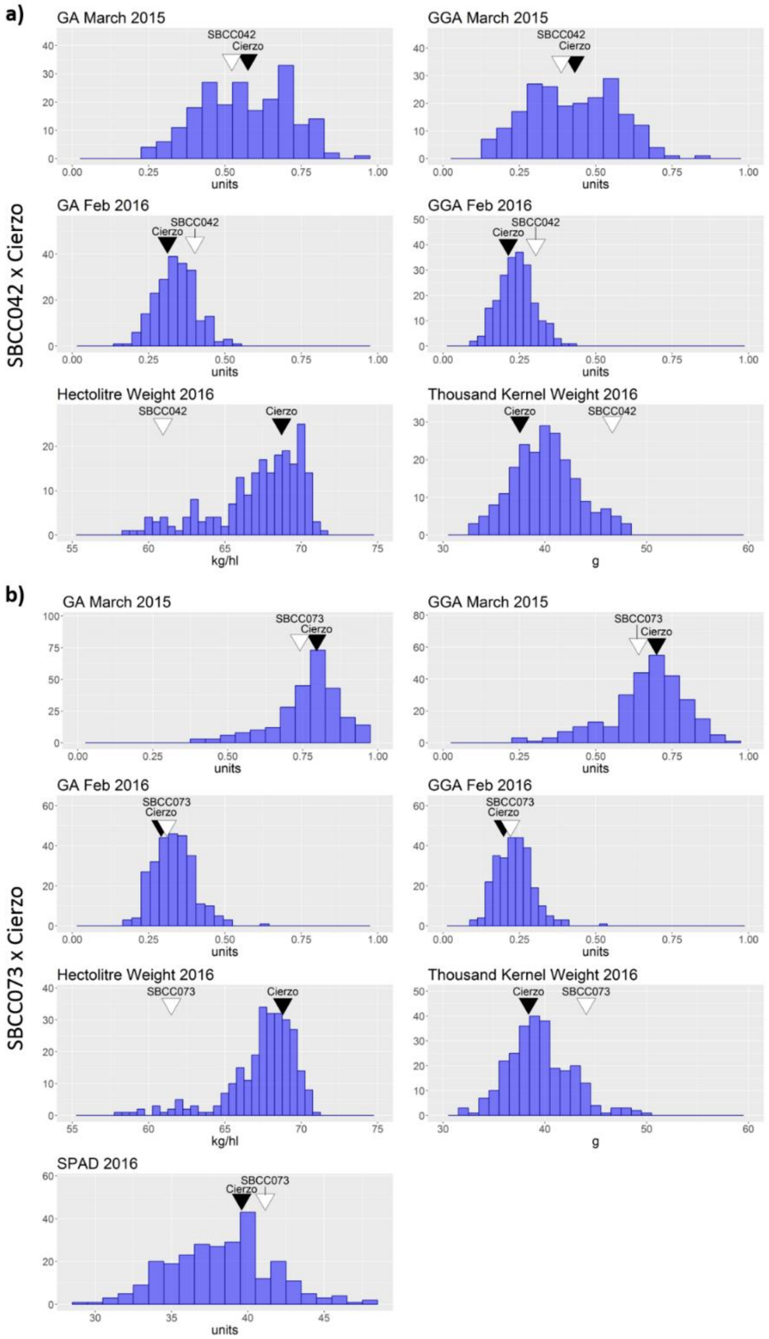

Figure S3. Histogram of distribution of green and greener area (GA and GGA respectively) in two years and SPAD, thousand kernel weight, hectolitre weight, in one year, measured in both populations. a) SBCC042 x Cierzo. b) SBCC073 x Cierzo. GA and GGA measurements of 2015 correspond to March, and those of 2016 correspond to February. Cierzo, SBCC042 and SBCC073 represent an average of 28, 8 and 8 replicates, respectively.

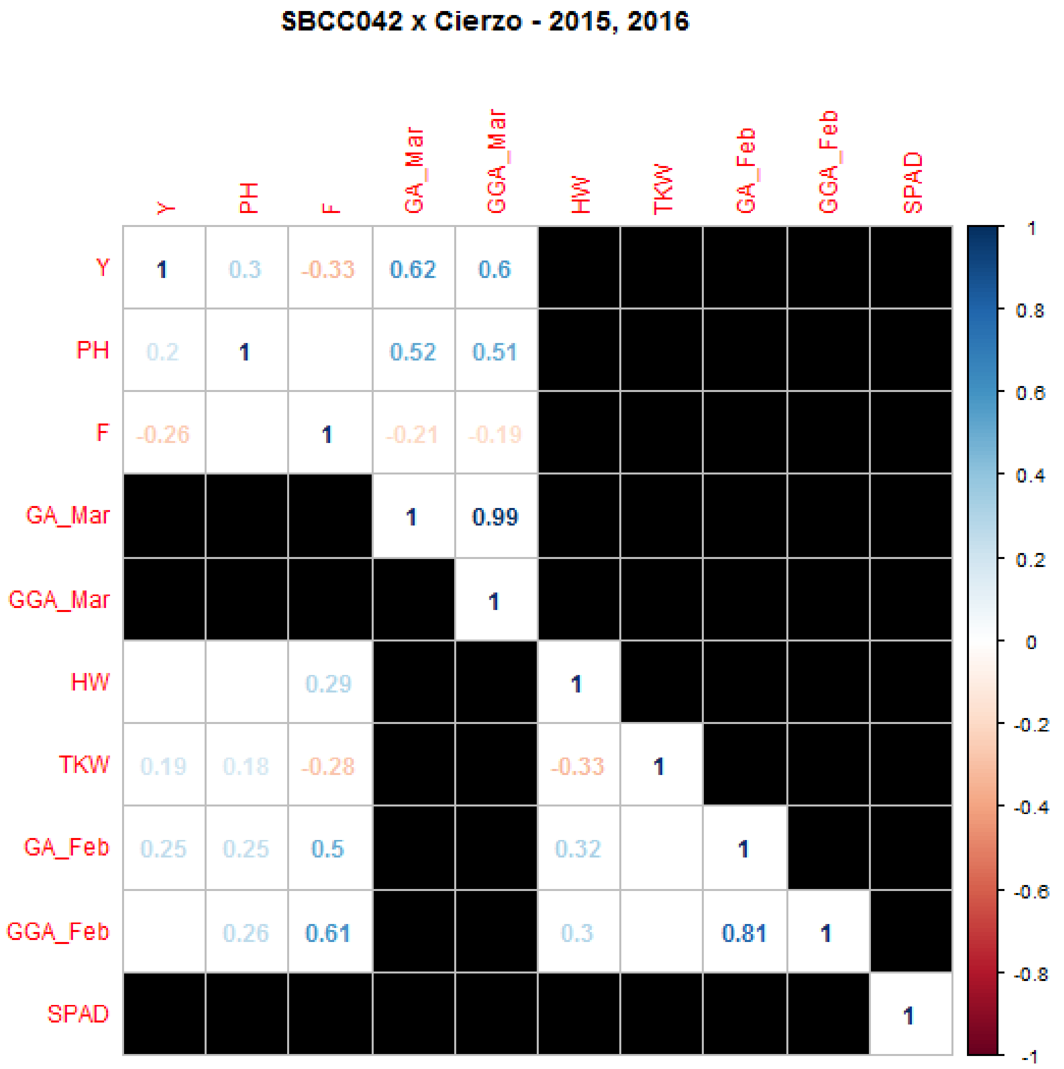

Fig S4. Pearson correlations between traits measured in the field for population SBCC042 x Cierzo. Upper triangle represents the traits recorded in 2015. Lower triangle represents the traits recorded in 2016. Empty cells are non-significant correlations (P<0.01). Black cells mean that there were no data for those traits.

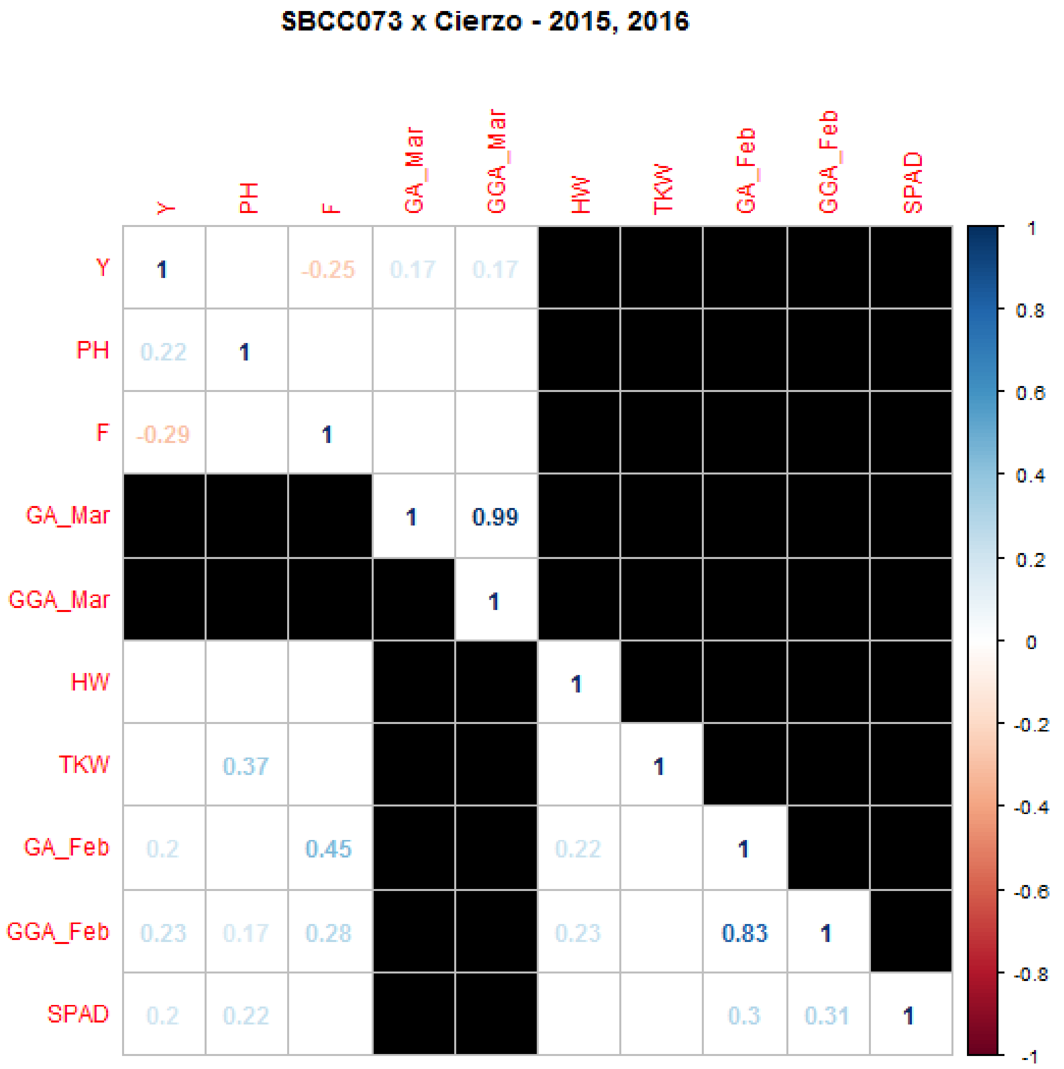

Fig S5. Pearson correlations between traits measured in the field for population SBCC073 x Cierzo. Upper triangle represents the traits recorded in 2015. Lower triangle represents the traits recorded in 2016. Empty cells are non-significant correlations (P<0.01). Black cells mean that there were no data for those traits.

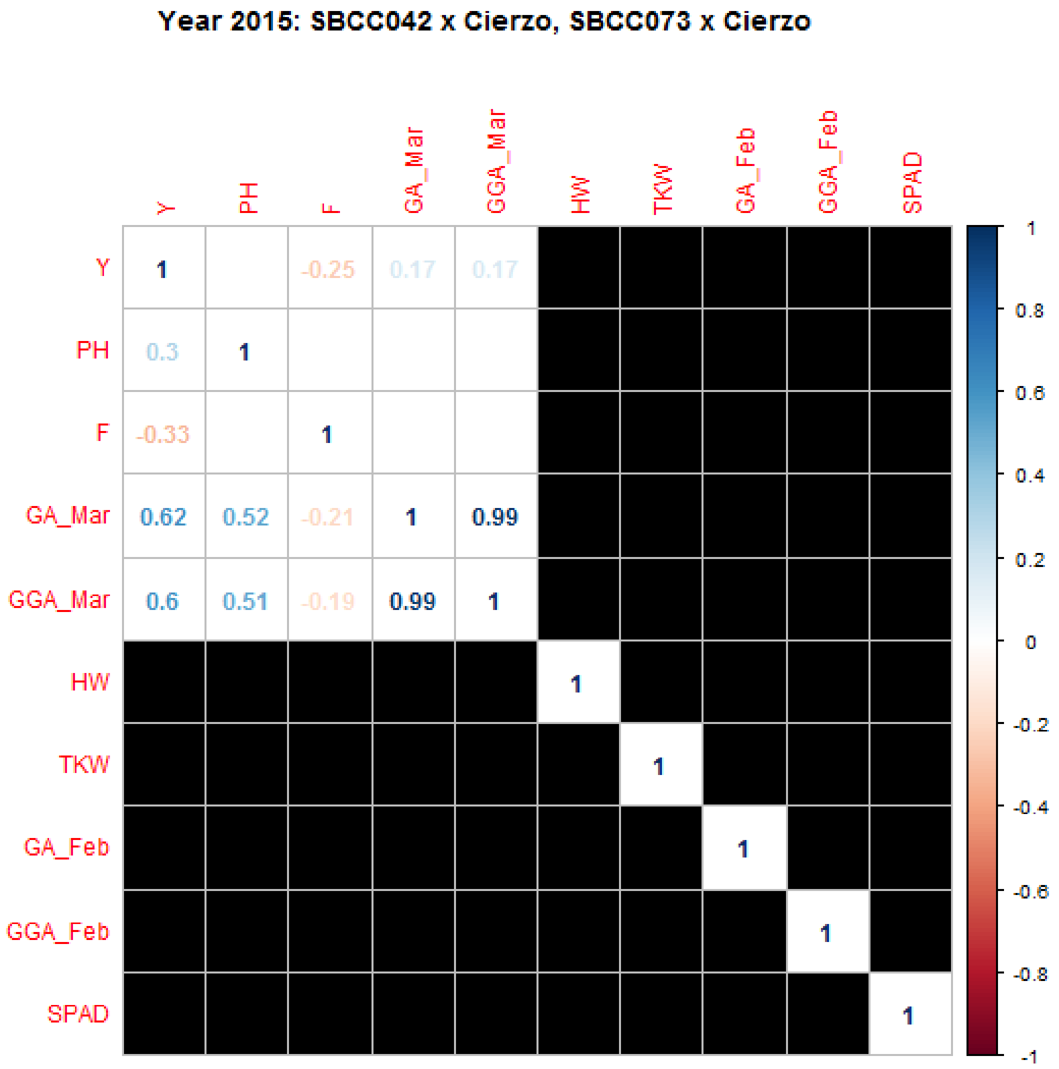

Fig. S6. Pearson correlations in 2015, within traits studied in both populations. The upper triangle represents the traits recorded in SBCC073 x Cierzo. The lower triangle represents the traits recorded in SBCC042 x Cierzo. Empty cells are non-significant correlations (P<0.01). Black cell means that there were no data for those traits.

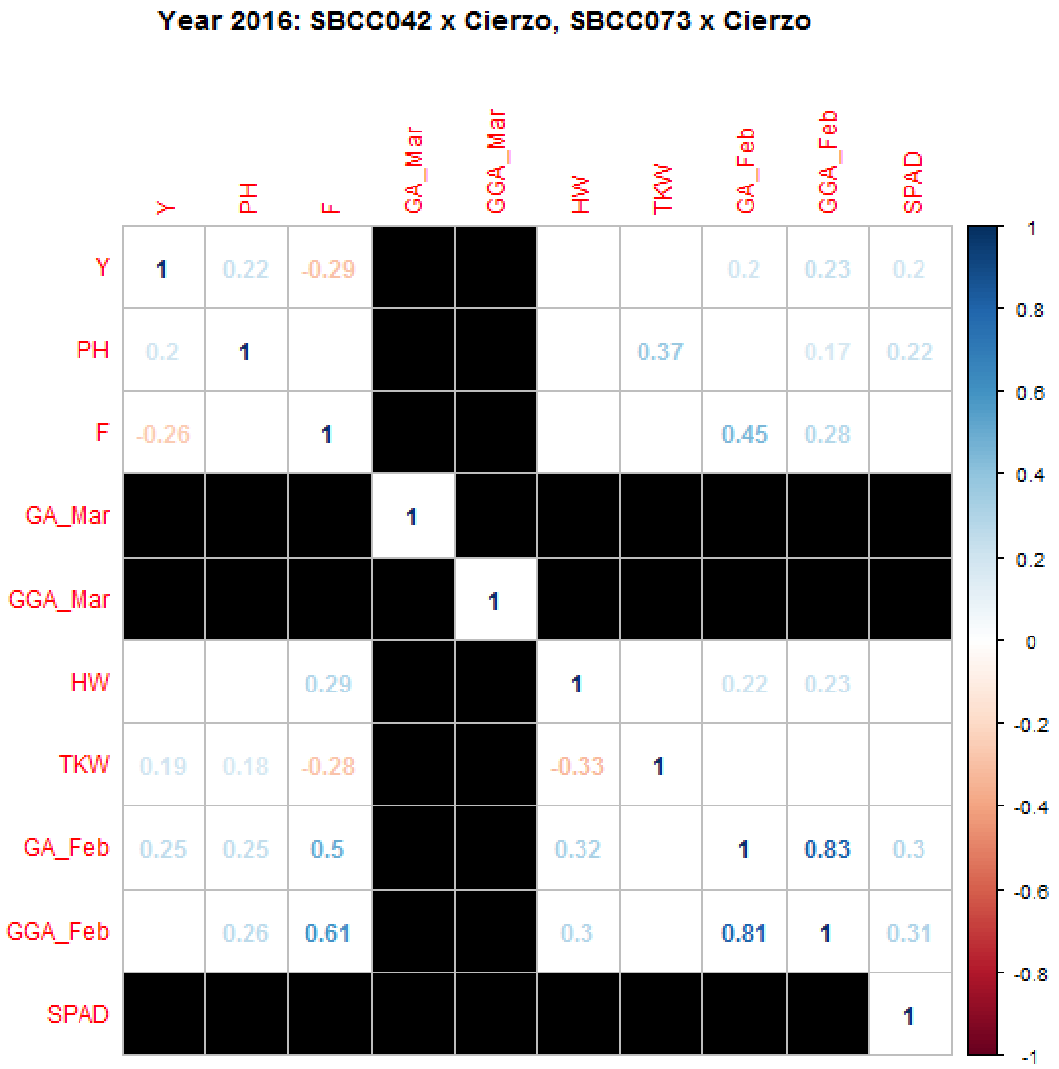

Fig.S7. Pearson correlations in 2016, within traits studied in both populations. The upper triangle represents the traits recorded in SBCC073 x Cierzo. The lower triangle represents the traits recorded in SBCC042 x Cierzo. Empty cells are non-significant correlations (P<0.01). Black cell means that there were no data for those traits.

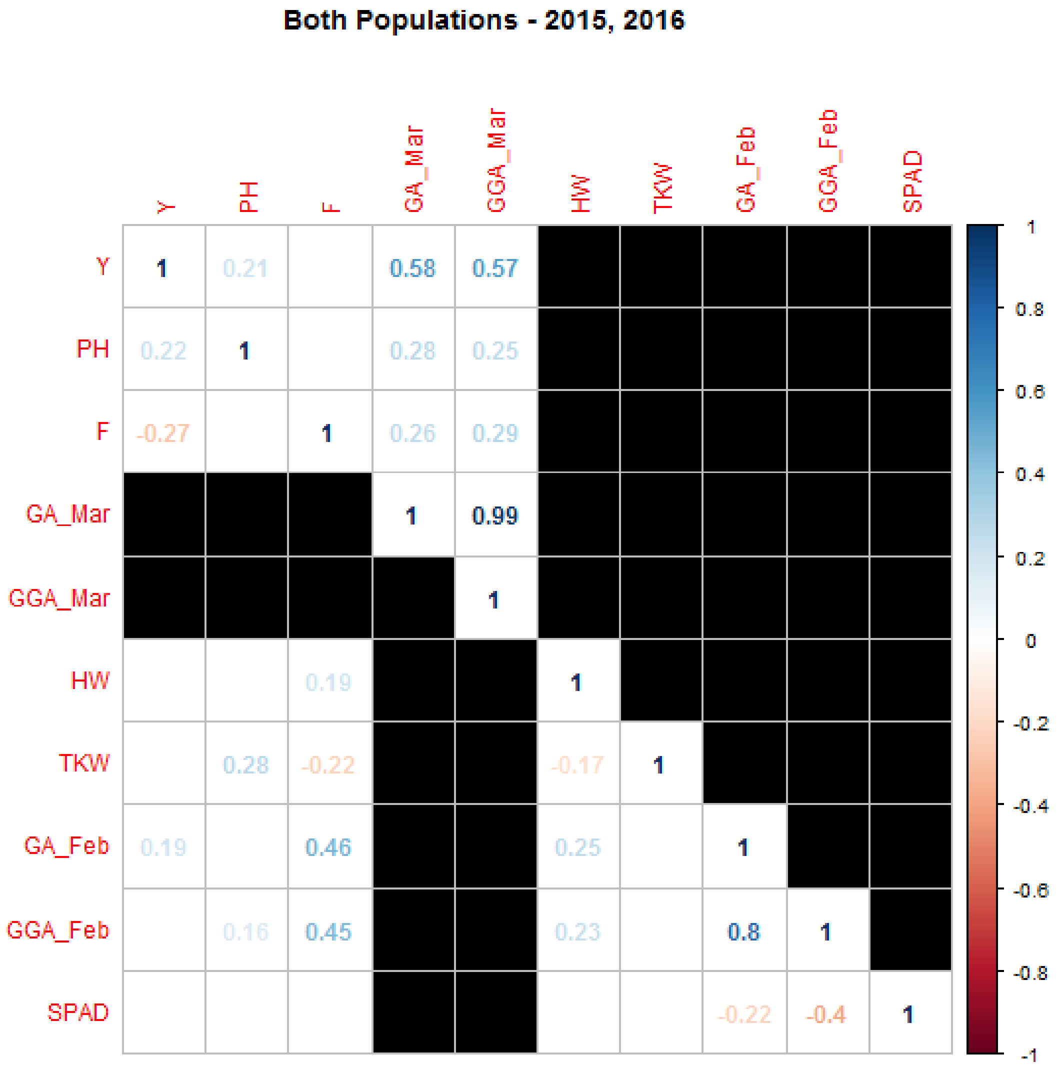

Fig.S8. Pearson correlations in two years (2015 and 2016) considering the two populations together. The upper triangle represents 2015; the lower triangle represents 2016. Empty cells are non-significant correlations (P<0.01). Black cells mean that there were no data for those traits.

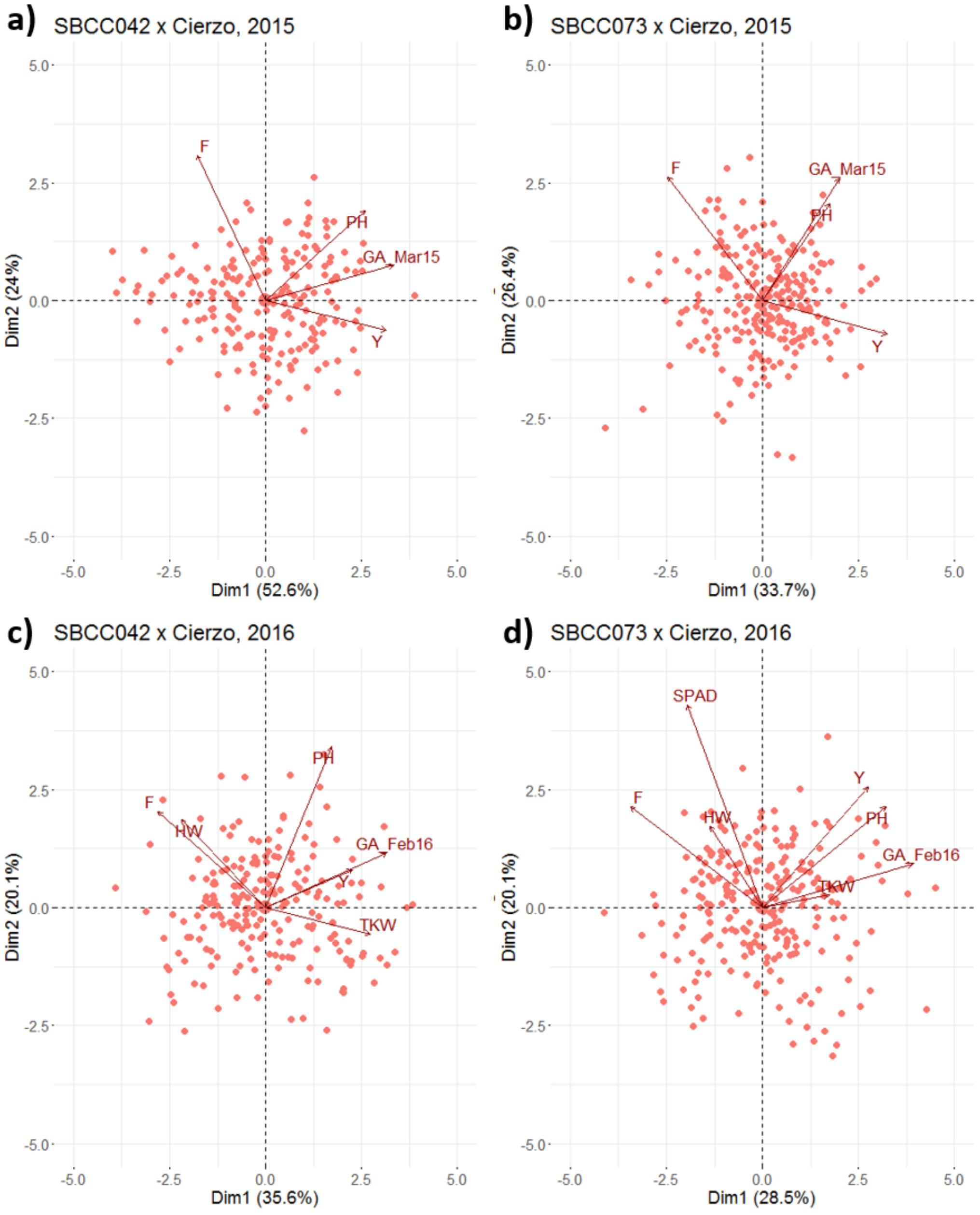

Figure S9. PCA biplot of the first two principal components calculated for all traits measured at each population in: a) and b) 2015, c) and d) 2016.

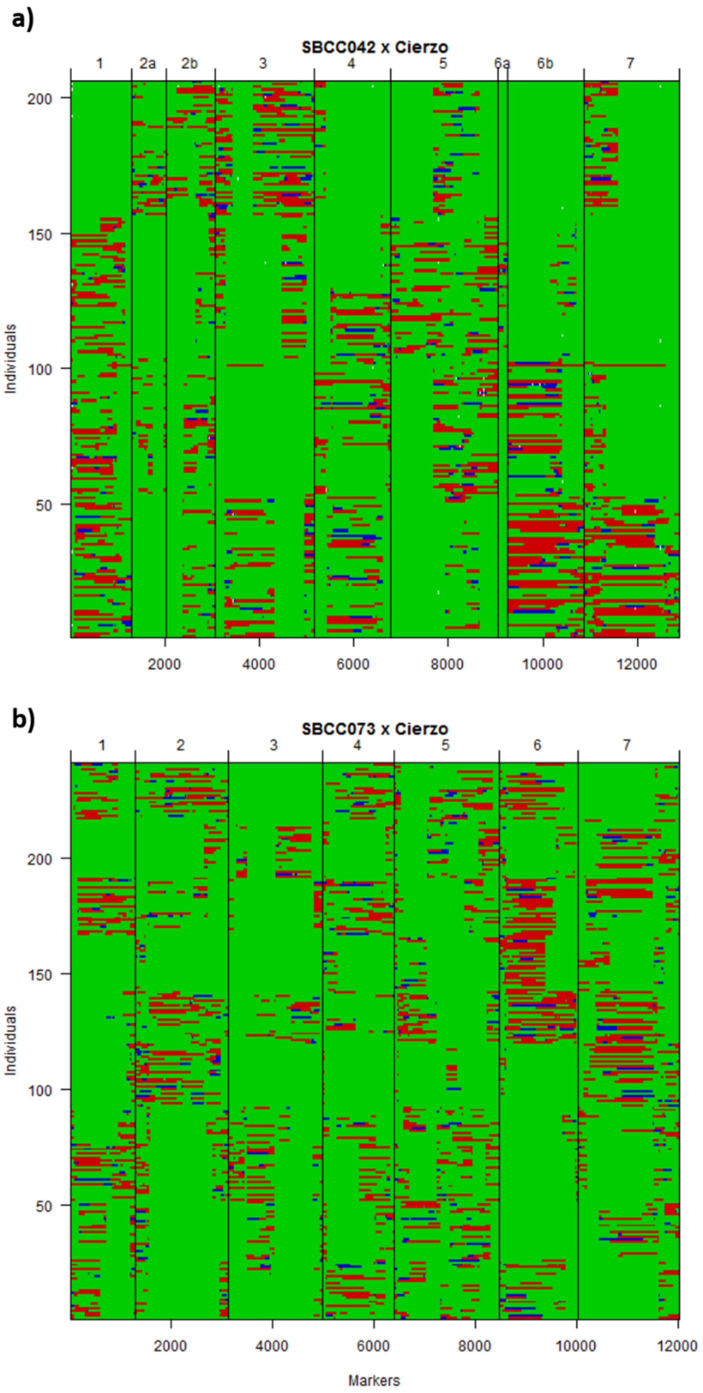

Fig. S10. Graphical genotypes. Cierzo allele in green; red, landrace allele; blue, heterozygote. Upper x-axis represents the linkage group; lower x-axis denotes the markers in sequential order; each line of y-axis corresponds to one BC2F5 line.

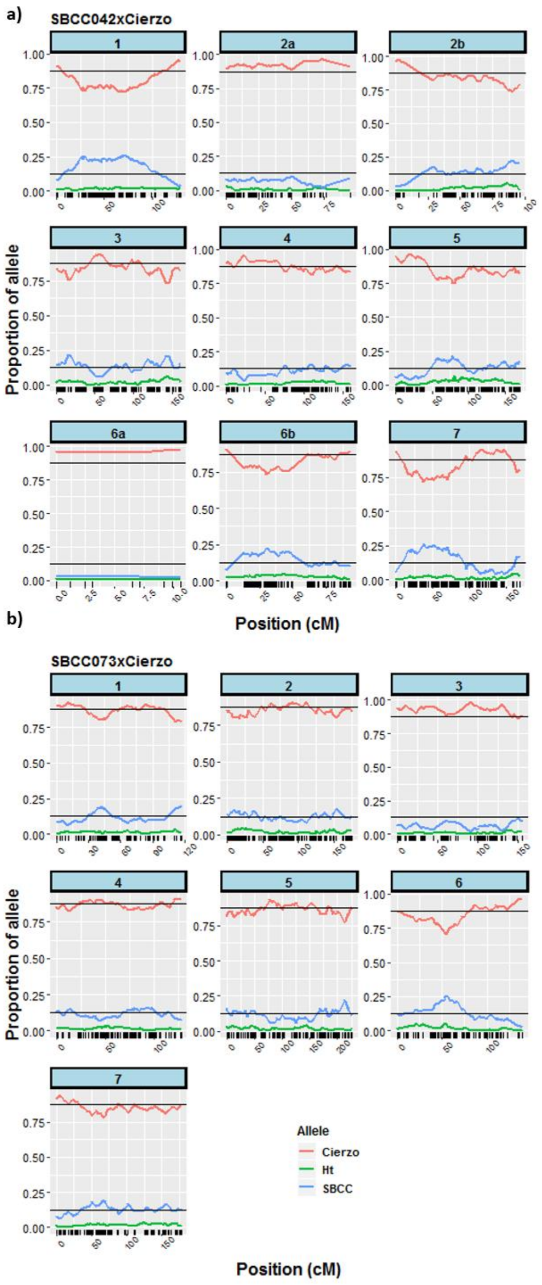

Figure S11. Proportion of alleles, distributed in the genetic map. Red lines represent the proportion of the alleles from parent Cierzo; green lines represent heterozygotes (Ht), and blue lines represent the SBCC parent allele: a) SBCC42; b) SBCC073. Each linkage group is represented (blue box) with the genetic positions in the corresponding map (black lines at the x-axes).

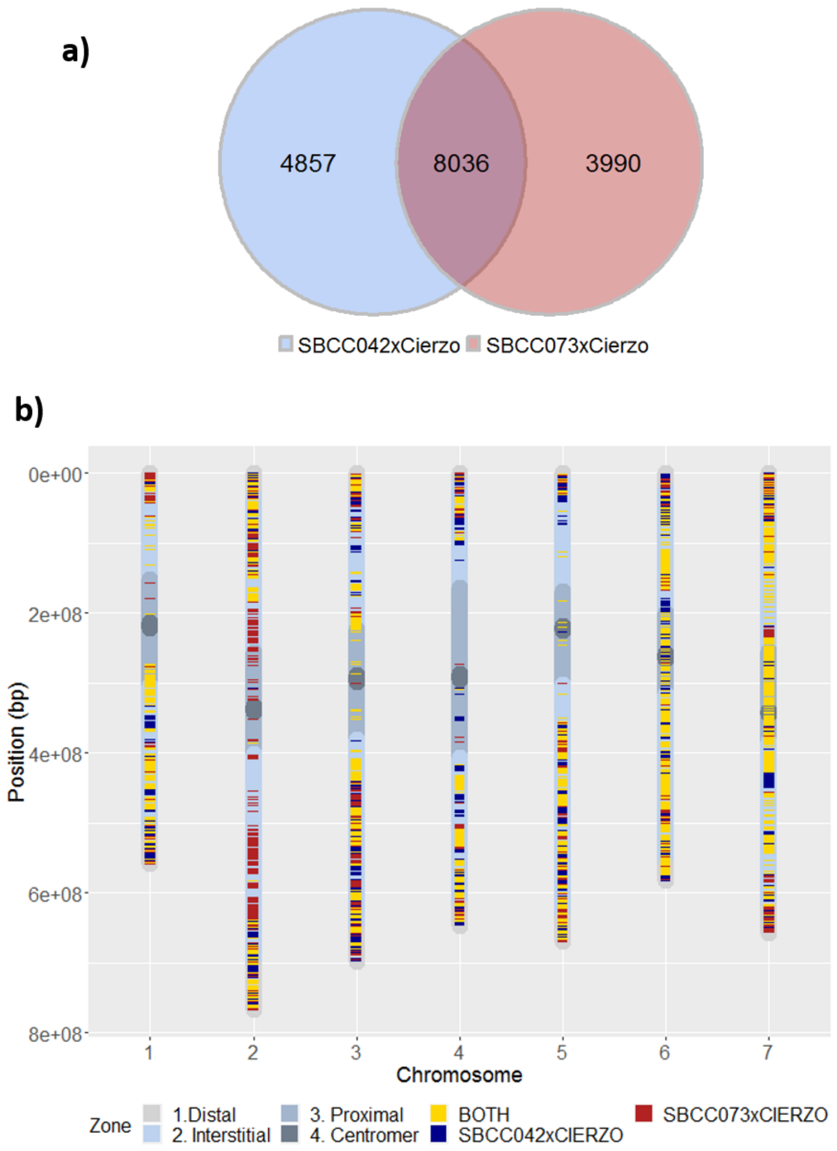

Figure S12. Shared and unique markers in the populations studied. a) Venn diagram with the number of polymorphic markers obtained for both populations. b) Positions in the physical map (Mascher et al., 2017). The chromosome segment represents each of the regions (distal in light grey, interstitial in light blue, proximal in grey and centromere in dark grey). The coloured lines across the chromosome represent the presence of a polymorphic marker in both populations (yellow), only in SBCC042 x Cierzo (blue) and only in SBCC073 x Cierzo (red).

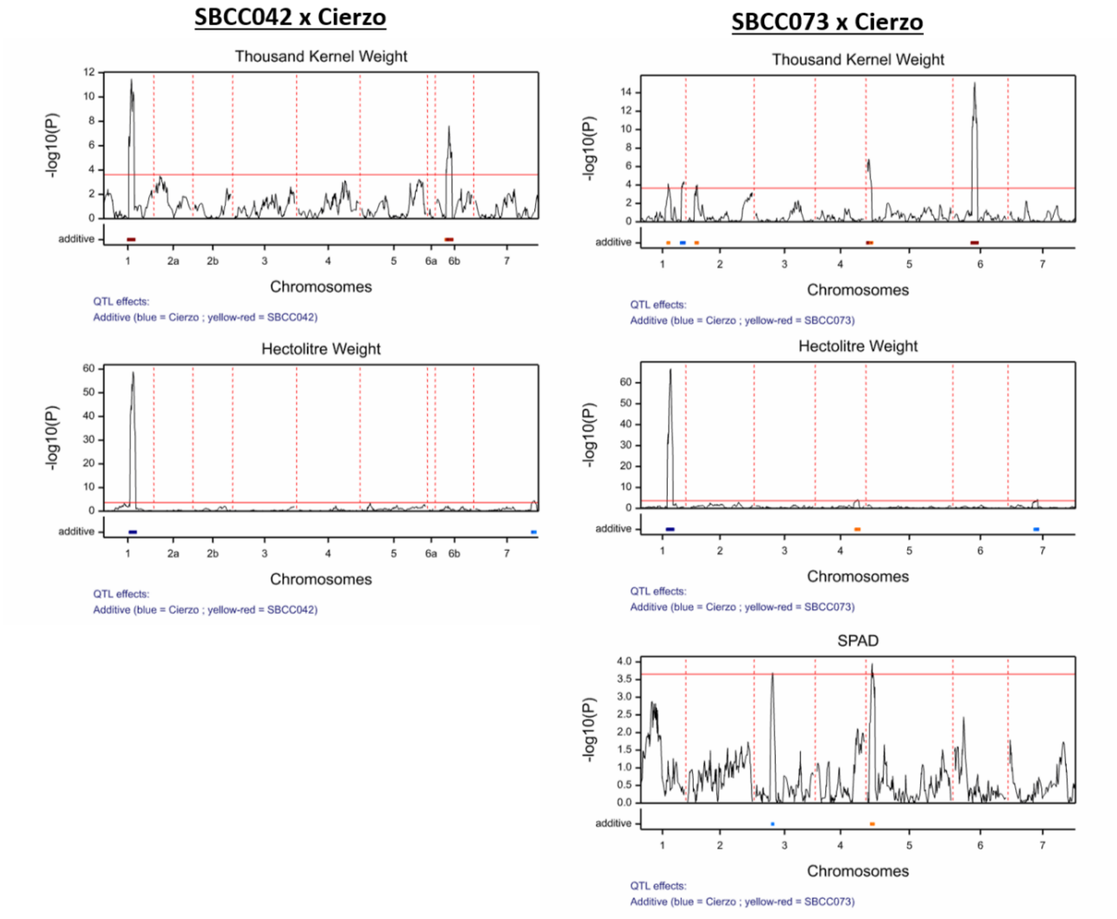

Figure S13. QTL scans for thousand kernel weight (TKW), hectolitre weight (HW) and SPAD score in 2016 in both populations, SBCC042 x Cierzo on the left, SBCC073 x Cierzo on the right.

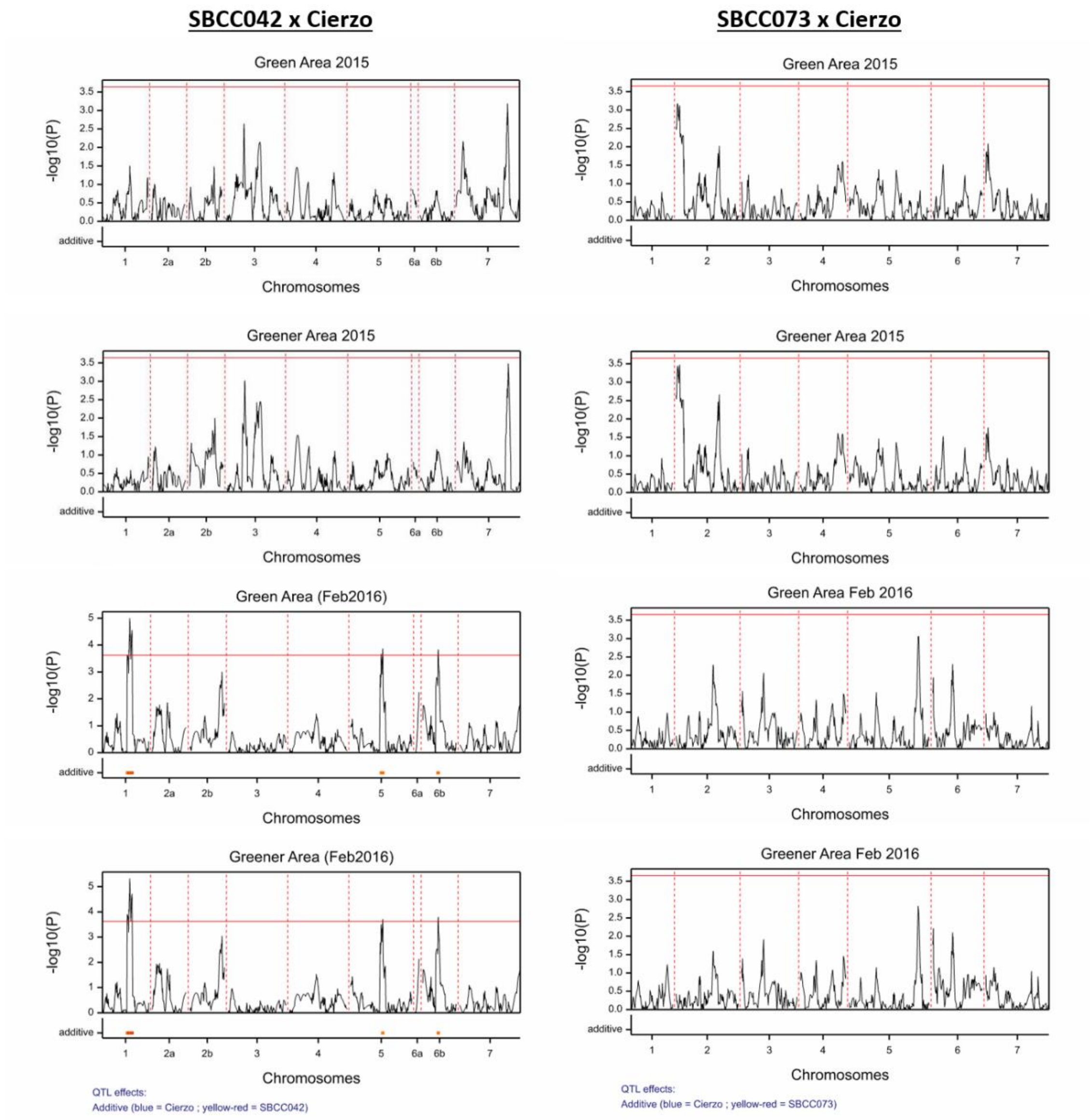

Figure S14. QTL scans of ground cover traits: green area and greener area. Ground cover was measured in March in 2015 and in February in 2016.

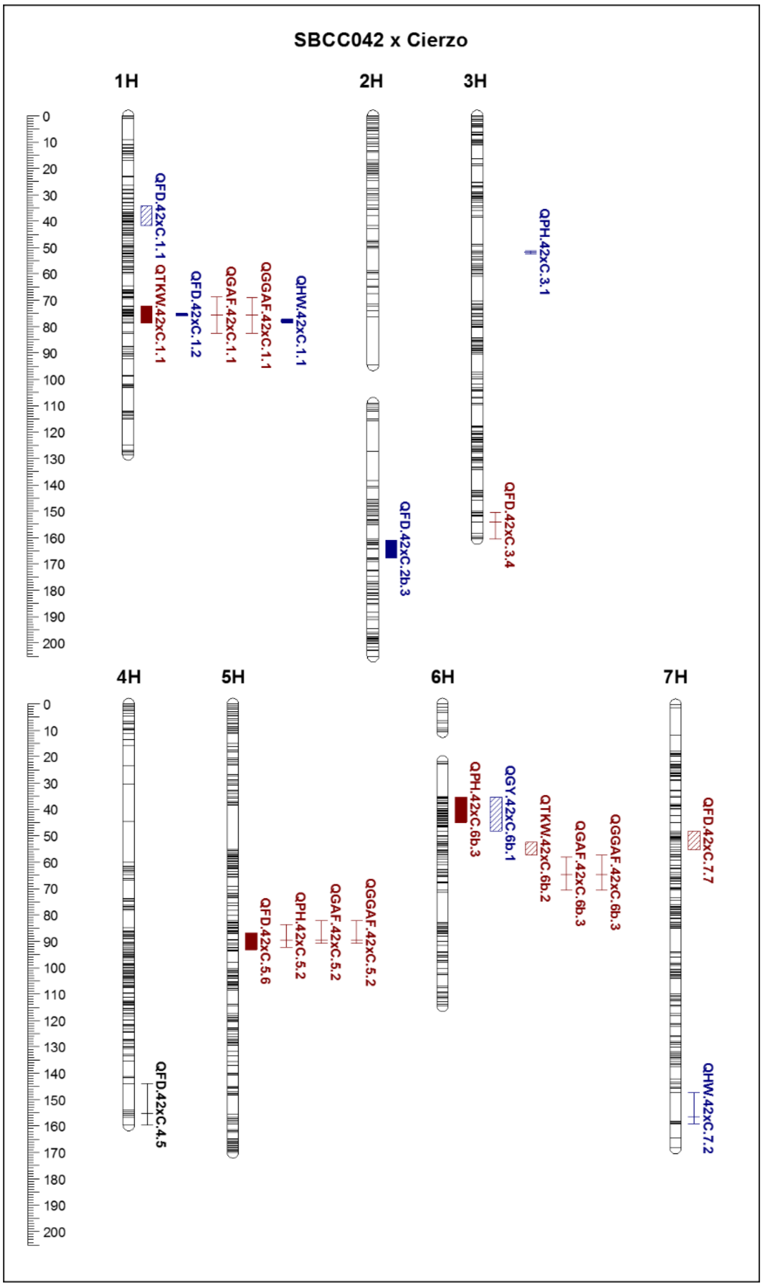

Figure S15. SBCC042 x Cierzo genetic map. Blue segments represent the QTL region for which Cierzo is associated with higher phenotypic value; red segments represent the QTL for which the SBCC042 allele is associated with higher phenotypic value. Shape of the segment represents strength of the QTL effect: line, 3.605 < -log10(P) < 6; diagonal hatch, 6.001 < -log10(P) < 10; filled box, -log10(P) > 10.

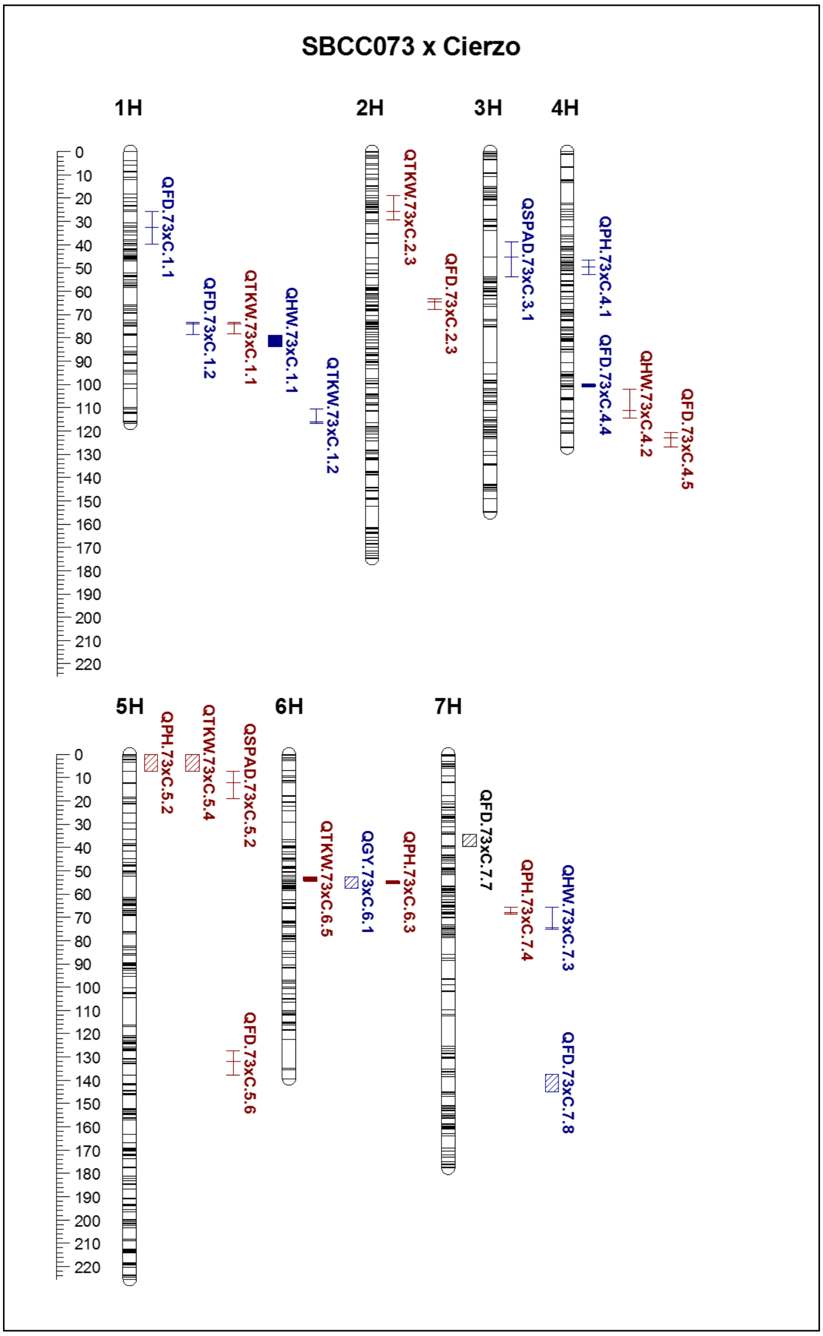

Figure S16. SBCC073 x Cierzo genetic map. Blue segments represent the QTL region for which Cierzo is associated with higher phenotypic value; red segments represent the QTL for which the SBCC073 allele is associated with higher phenotypic value. Shape of the segment represents strength of the QTL effect: line, 3.605 < -log10(P) < 6; diagonal hatch, 6.001 < -log10(P)<10; filled box, -log10(P) > 10.

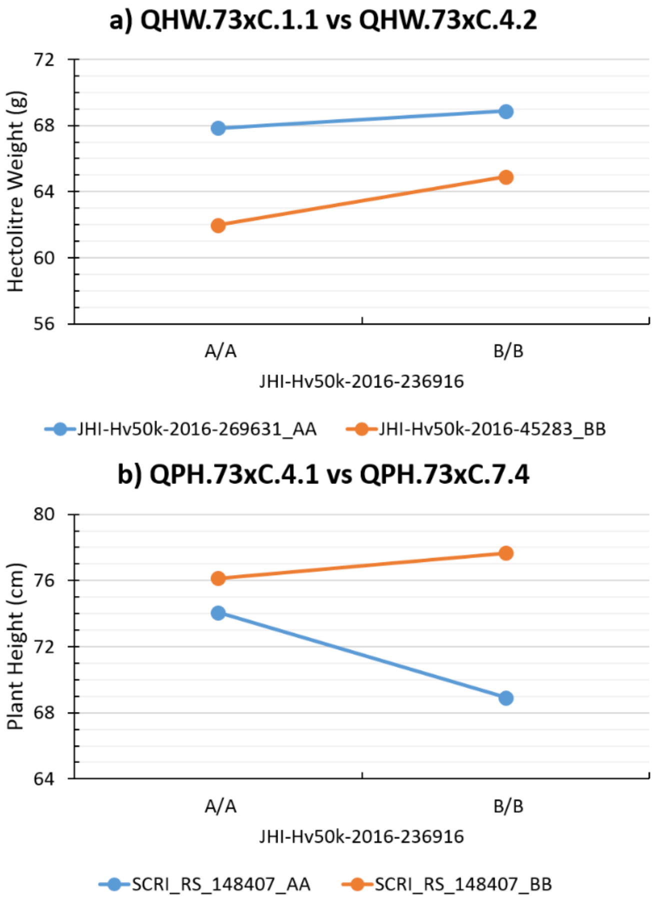

Figure S17. Representation of the two significant QTL interactions found in the pairwise analyses of variance, considering the homozygous alleles only (when each genotype class was represented by more than 5 individuals). The x-axis denotes the alleles for the first marker (Cierzo as AA, SBCC073 as BB). The y-axis indicates the value for the trait analysed. Each allele for the second marker is represented in blue dots for Cierzo alleles, and orange dots for SBBC073 alleles.
